# Characterization of the virulence of three novel clade 2 *Clostridioides* (*Clostridium*) *difficile* strains and a two-year screening in animals and humans in Brazil

**DOI:** 10.1371/journal.pone.0273013

**Published:** 2022-08-26

**Authors:** Amanda Nadia Diniz, Loren Nery Fontoura Moura, Diogo Soares Gonçalves Cruz, Carlos Augusto Oliveira Junior, Henrique César Pereira Figueiredo, João Luís Reis Cunha, Eduardo Garcia Vilela, Edward J. Kuijper, Mark H. Wilcox, Francisco Carlos Faria Lobato, Rodrigo Otávio Silveira Silva

**Affiliations:** 1 Department of Preventive Veterinary Medicine Federal University of Minas Gerais (UFMG), Belo Horizonte, Minas Gerais, Brazil; 2 Clinic Hospital of Federal University of Minas Gerais, Belo Horizonte, Minas Gerais, Brazil; 3 Expertise Center for Clostridioides difficile infections, at Department of Medical Microbiology, Leiden University Medical Center, Leiden, and the National Institute for Public Health and the Environment, Bilthoven, The Netherlands; 4 Institute of Biomedical and Clinical Sciences, University of Leeds, Leeds, United Kingdom; Cornell University, UNITED STATES

## Abstract

*Clostridioides* (*Clostridium*) *difficile* infection (CDI) is an evolving global healthcare problem, and owing to the diverse and dynamic molecular epidemiology of *C*. *difficile*, new strains continue to emerge. In Brazil, only two cases of CDI due to the so called hypervirulent PCR ribotype (RT) 027 belonging to clade 2 have ever been reported, whereas incidence of CDI due to another “hypervirulent” RT078 (clade 5) has not yet been reported. In contrast, novel clade 2 strains have been identified in different hospitals. To better understand the epidemiology of CDIs in Brazil, this study aimed to genotypically and phenotypically characterize three novel Brazilian clade 2 strains (RT883, 884, and 885) isolated from patients with confirmed CDI. In addition, to better understand the circulating RTs, a two-year sampling was conducted in patients from the same hospital and in several domestic and wild animal species. The three strains examined showed lower production of A/B toxins than the control RT027, although two of these strains harbored a truncated *tcdC* gene. All strains showed swimming motility similar to that of RT027, while RT883 showed higher spore production than the reference strain. In the *in vivo* hamster model, the lethality of all strains was found to be similar to that of RT027. Both cgMLST and cgMLSA analyses revealed a high genetic similarity among the three-novel clade 2 isolates. In the two-year survey in animals and humans, RT883, 884, and 885 were not detected; however, three new RTs (RT988, RT989, and RT990) were isolated, two of which were genetically related to the three previously reported clade 2 strains. RT106 and RT126 were most frequently detected in humans (47.9%) and animals (57.9%), respectively. Furthermore, RT027 and RT078 were not detected in humans. The results of this study suggest that these novel clade 2 strains have virulence potential and that new strains from clade 2 continue to emerge in our setting, indicating the need for long-term local surveillance.

## Introduction

*Clostridioides* (prev. *Clostridium*) difficile is a major cause of healthcare-associated infections and is responsible for an increasing number of community-acquired diarrhea cases worldwide [1]. *Clostridioides difficile* infection (CDI) gained significant attention in the early 2000s, when the incidence and severity of the disease increased in several countries due to the emergence of epidemic strains [2]. These strains, also previously called “hypervirulent” strains, are commonly classified into ribotypes (RTs) 027 and 078 and into clades 2 and 5 through multilocus sequence typing (MLST). Following this marked epidemiological change in CDI, studies have revealed the emergence of other strains from clade 2 and those related to RT027, such as RT176, in eastern Europe [2, 3] and RT244 and RT251 in Australia [4, 5]. Thus, owing to the highly dynamic epidemiology of CDI, it is believed that the emergence of new epidemic strains is likely to occur continuously [4–7].

Studies suggest that RT027 and RT078 are extremely rare in humans in Brazil [8–10]. The absence or rare isolation of both RT027 and RT078 contrasts with reports in North America and most European countries but is consistent with previous studies reported in China that have suggested the emergence of other potentially epidemic genotypes of *C*. *difficile*, including strains from clade 3 [11]. To date, RT106 and 014/020 have been identified as the most frequent *C*. *difficile* strains in both humans and animals in Brazil [9, 10, 12, 13], whereas RT027 has only been described in two individuals in the southern region of Brazil [14]. In contrast, the circulation of novel isolates from clade 2 in hospitals in Brazil poses a risk of emergence and spread of new epidemic strains, such as the recently reported RT181 in Greece [9, 15–17]. Recently, we described strains from new RTs and new sequence types (RT883/ST461, RT884/ST462, RT885/ST463), all classified into clade 2, at the Clinical Hospital of Universidade Federal de Minas Gerais [9]. One of these strains has also been recently reported in a hospital in Rio de Janeiro [16]; this finding confirms the circulation of this novel strain in hospitals in Brazil.

The present study aimed to phenotypically and genotypically characterize three novel clade 2 strains isolated from patients with CDI in a university hospital in Belo Horizonte, Brazil. In addition, in order to evaluate the occurrence of novel epidemic strains, samples from several animal species and from inpatients were subjected to isolation of *C*. *difficile* and ribotyping during a 24-month period.

## Material and methods

### *Clostridioides difficile* isolates used in genotypic and phenotypic characterization

Between 2011 and 2017, studies were conducted at the Clinical Hospital of the Federal University of Minas Gerais (HCUFMG) with in inpatients with confirmed CDI [9]. The HCUFMG is a 400-bed quaternary care hospital located in Belo Horizonte, Minas Gerais State, southeast Brazil [9]. During these studies, three strains from clade 2, classified as new RTs and STs (RT883/ST461, RT884/ST462, RT885/ST463) were isolated (Table 1). These strains were found in inpatients with confirmed CDI, all positive for free A/B toxins in stools and two with severe CDI according to the Infectious Diseases Society of America (IDSA) and Society for Healthcare Epidemiology of America (SHEA) criteria [18].

**Table 1:** Sequence type, ribotyping, year of isolation, and presence of the main toxin genes in the three Brazilian clade 2 strains isolated between 2011 and 2015 in Belo Horizonte, Minas Gerais.

Strain	Year	Ribotype	Multilocus sequence type		Toxin genes	Reference	Genome Accession Number
			Sequence type	Clade			
**HC27/15**	2015	883	461	2	A+B+CDT+	[9]	GCA_023656905.1
**HC58**	2012	884	462	2	A+B+CDT+		GCA_023656875.1
**HC76**	2013	885	463	2	A+B+CDT+		GCA_023656865.1

### Determination of TcdA/TcdB production

Free toxin levels produced in vitro were determined at two different time points (24 and 72 h post inoculation), as described previously [19]. Briefly, pure aliquots from each strain were plated onto Müller Hinton agar (Oxoid, UK) supplemented with 7% horse blood and 0.1% sodium taurocholate (Sigma-Aldrich Co., USA), followed by anaerobic incubation at 37°C for 18–24 h (stationary phase). After incubation, a suspension of *C*. *difficile* was prepared in sterile 0.85% saline, using McFarland standard 1; 100 μL of the suspension was transferred to cryotubes containing 500 μL of BHI broth (Oxoid, UK), and the tubes were subjected to anaerobic incubation at 37°C. After incubation for 24 and 72 h, the culture was centrifuged at 16,000 × g for 10 min at 4°C and filtered through a membrane filter with a 0.22 μm pore size. The toxin concentrations in the supernatants were evaluated using a commercial enzyme immunoassay (Ridascreen *C*. *difficile* Toxin A/B® - R-Biopharm, Germany) and the absorbance was measured at 450 nm according to the manufacturer´s instructions. Strain CD196 (RT027) was used as the reference strain.

### Sporulation assay

Spore production and spore quantification were performed according to previously described protocols [20, 21]. Briefly, pure aliquots from each strain were plated onto Müller Hinton agar (Oxoid, UK) supplemented with 7% horse blood and 0.1% sodium taurocholate (Sigma-Aldrich Co., USA), followed by anaerobic incubation at 37°C for 48 h. After incubation, a suspension of *C*. *difficile* was prepared in sterile 0.85% saline using McFarland standard 1 as the reference. Thereafter, the suspension was diluted in fresh, pre-reduced BHIS medium at a 1:1000 ratio, followed by anaerobic incubation at 37°C for 72 or 120 h. After incubation, each suspension was thermally treated for 20 min at 70°C, and serial dilutions (10–2 to 10–5) were plated onto Müller Hinton agar (Oxoid, UK) supplemented with 7% horse blood and 0.1% sodium taurocholate (Sigma-Aldrich Co., USA) and incubated under anaerobic conditions at 37°C for 48 h. The isolate CD196 (RT027) was used as the reference strain. Tests were performed in sextuplicate for each strain during the two periods evaluated (72 h and 120 h).

### Motility assay

Motility assays were performed to compare the swimming behavior of *C*. *difficile* strains according to previous studies [19]. Briefly, motility plates were prepared by adding 25 mL of BHI broth (Oxoid, UK) with 0.3% (w/v) bacteriological agar (Micromed, Brazil). The agar plates were transferred to an anaerobic chamber for at least 4 h prior to inoculation. For each strain, a suspension of *C*. *difficile* was prepared in sterile 0.85% saline (McFarland standard 1), and 3 μL of the suspension was inoculated in the middle of the plate, which was then incubated at 37°C in anaerobiosis. Motility was quantitatively determined by measuring the diameter of the zone of motility at three different time-points (24, 48, and 72 h). The isolate CD196 (RT027) was used as the reference strain. Motility assays were performed in triplicate for each strain, and each assay was repeated three times independently [19].

### Antimicrobial susceptibility

Minimum inhibitory concentrations (MICs) for metronidazole, vancomycin, rifampicin, clindamycin, erythromycin, moxifloxacin, and tetracycline were determined using a gradient test with ETEST (bioMérieux Brazil, France) according to the manufacturer’s instructions. Bacteroides fragilis (ATCC 25285) and *Staphylococcus aureus* (ATCC 25923) were used as controls. The epidemiological cut off value for vancomycin and metronidazole recommended by the European Committee on Antimicrobial Susceptibility Testing (EUCAST) was used. Isolates with rifampin MIC ≥32 mg/mL were defined as rifampin resistant [22], and the breakpoints for clindamycin, erythromycin, moxifloxacin, and tetracycline were based on Clinical & Laboratory Standards Institute (CLSI) recommendations [23].

### Survival analysis in hamster model

The hamster model with Golden Syrian hamsters (*Mesocricetus auratus*) was based on previously described protocols with some modifications [24–26]. Six- to eight-week-old, male Syrian hamsters with a body weight of 85–120 g were used. Similar to previous studies, five batches of spores per *C*. *difficile* strain were produced as previously described [20]. Thirty-six animals, in individual cages, were randomly separated into five groups of six animals (I–V). All animals received 30 mg kg-1 of clindamycin intramuscularly. After 48 h, animals from groups I to IV received 100 μL (containing 10^4^ colony forming unit (CFU) spores) of the control reference strain (RT027/CD196), RT883, RT884, and RT885 strains by esophageal gavage, respectively. Hamsters from group V were subjected to gavage with 0.85% saline for 48 h. All animals were monitored, by trained veterinarians, every 4 h for 15 days for signs of infection, such as weight loss and wet tail (diarrhea), and to record their mortality [27]. Animals considered moribund were immediately submitted to euthanasia [28]. During necropsy, cecum of all animals was collected and subjected to A/B toxin detection using a commercial enzyme-linked immunosorbent assay kit (Ridascreen *C*. *difficile* Toxin A/B®, R-Biopharm, Germany) according to the manufacturer’s recommendations. In addition, the fecal samples were submitted for isolation of *C*. *difficile*, followed by detection of *tpi*, *tcdA*, *tcdB*, and *cdtB* [29] and PCR ribotyping [30] to confirm the match with the inoculated strains from each group.

### Whole-genome sequencing

The three clade 2 strains (RT883/ST461, RT884/ST462, RT885/ST463) were grown on Mueller–Hinton agar supplemented with 5% blood and 0.1% taurocholate at 37°C under anaerobic conditions for 48–72 h. Genomic DNA was extracted using the Maxwell 16® Research Instrument (Promega, USA) combined with lysozyme (10 mg/mL) and proteinase K (20 mg/mL). Briefly, cells were incubated overnight in a lysozyme solution (10 mg/mL) at 37°C, followed by addition of proteinase K and incubation at 56°C for 30 min. According to the kit instructions: (i) the samples were lysed in the presence of a chaotropic agent and detergent; (ii) the nucleic acids were bound to silica magnetic particles; (iii) the bound particles were washed to isolate them from other cell components; and (iv) the nucleic acids were eluted into a formulation for sequencing. The extracted DNA was stored at −80°C until analysis. The genome was sequenced using the Ion Torrent PGM™, in a mate-pair sequencing kit with an insert size of 3 kbp (~144-fold coverage) and with a fragment sequencing 400 bp kit (~318-fold coverage). The quality of the raw data was analyzed with FastQC (http://www.bioinformatics.babraham.ac.uk/projects/fastqc). The reads were polished with Trimmomatic [31], retaining only paired reads with phred quality of 30 or higher and minimal size of 50 nucleotides. BWA-mem and Samtools [32] were used to assess the genome coverage by mapping the reads to the *C*. *difficile* reference strain CD630 (GenBank: NC_009089.1). The genome of the evaluated isolates was assembled with SPAdes 3.5.0 in the careful mode [33], using only paired-end library, and the construction of a super scaffold was performed with the CONTIGuator 2.0 software [34], using the default parameters and *C*. *difficile* strain CD630 (GenBank: NC_009089.1) as the reference. Finally, GAP filling and polishing were performed using Pilon [35].

#### Comparative genomics

Average nucleotide identity analysis between the three isolates was performed using FastANI [36], and the phylogenetic network was estimated with the Splittree4 program [37] using the Neighbournet method. The visual comparison of RT883, RT884, and RT885 with reference genomes from different clades, including clade 2, was performed using BRIGS [38]. Phage were identified using PHASTER [39] and ProphET [40]. Transposons were identified through manual inspection of the annotation and gene structure of the genomic regions aligned regions using progressive Mauve [41]. In silico PCR was performed to search for additional virulence factors and antimicrobial resistance genes using Ipcress (https://www.ebi.ac.uk/about/vertebrate-genomics/software/ipcress-manual). In addition, the CARD portal [42] and Resfinder [43] were used to identify antimicrobial resistance genes. Paloc and CdtLoc structures of RT883, RT884, and RT885 were compared with those of eight reference *C*. *difficile* strains: M120 (GCF_000210435.1), CD630 (GCF_000009205.2), M68 (GCF_000210395.1), CF5 (GCA_000210415.1), R20291 (GCA_000027105.1), 2007855 (GCA_000210455.1), Bl1 (GCA_000211235.1), and CD196 (GCF_000085225.1). The pan and core genome of *C*. *difficile* comprising the three isolates RT883, RT884, RT885 and the eight reference strains were obtained using Roary v3.13.0 [45]. Each sequence was annotated with Prokka v1.11 [46], using CD630 proteins as model, kingdom bacteria, genus *Clostridioides* and rRNA prediction with RNAmmer. Roary was used to assess the pan and core genome of the evaluated bacteria, with default parameters and the option -e and—mafft to generate the core genome multiple alignment. The pan-gene presence/absence gene plot was generated in R, using the function heatmap2 from the gplots library (https://cran.r-project.org/web/packages/gplots/index.html), clustering the isolates and genes by UPGMA from their Manhattan distances. The cgMLSA Maximum Likelihood phylogeny was performed using the alignment generated by Roary (2.643 genes, containing 2,531,373 positions) and the IQTRee v2.1.1 program [44], using the substitution model GTR+F+R5 selected as the best fit model by ModelFinder [45] and 1000 ultrafast bootstrap replicates [46]. The tree image was generated with ITOL online, with midpoint rooting [47].

Genes of interest (*tcdA*, *tcdB*, *tcdC*, *gyrA*, and *gyrB*) were submitted as query sequences and newly identified allele sequences were deposited in the PubMLST *C*. *difficile* database. A Bayesian phylogeny of *tcdB* was performed to compare all the alleles deposited in the database using Mr. Bayes v 3.2.7 [48]. The best model indicated by the Modeltest-NG was GTR + I. The phylogeny considered 5.000.000 MCMC generations, and the tree was visualized with ITOL (https://itol.embl.de/upload.cgi). Transposons were searched using BLASTn v.2.5.0 (https://blast.ncbi.nlm.nih.gov/Blast.cgi) and Nucmer (MUMmer v.3.0) [49]. The transposon genome localization figure for all isolates was generated using Circa, based on their genomic coordinates (http://omgenomics.com/circa). Additionally, the presence of partial or complete sequences of the main *C*. *difficile* transposons (AF109075, HG475346, KC166248, HG002387, HG002396, HG002395, HG002389, HG002386, AF333235, and AF226276) was investigated in the three *C*. *difficile* isolates, using BLASTn v.2.5.0 (https://blast.ncbi.nlm.nih.gov/Blast.cgi).

### Isolation of *C*. *difficile* from humans and animals (2018 to 2020)

To verify the RTs circulating in animals and humans, including the three novel Brazilian clade 2 or other possible epidemic strains, fecal samples from several animals and CDI patients were subjected to isolation of *C*. *difficile* between February 2018 and January 2020 (a total of 24 months). This study was approved by the Ethical Committee on Animal Use (CEUA) of the Federal University of Minas Gerais under the following protocols: 238/2015, 306/2017, 361/2018 and 213/2019, by Instituto Chico Mendes de Conservação da Biodiversidade (ICMBio) under protocols 49195–1 and 12989–2 (SISBIO 66535–2). All procedures involving humans were also previously approved by the Research Ethics Committee of the Faculty of Medicine of Federal University of Minas Gerais (CAAE—0710.0.203.0000.11).

#### Humans

In total, 152 stool samples from CDI patients were tested. The majority of these samples (143 samples, 94.1%) were obtained from inpatients of the HCUFMG and no outbreaks were observed during this period of time. The inclusion criteria for possible CDI cases were as follows: adults (18 years of age or older) who had received systemic antibiotics anytime in the last 3 months and presenting with acute diarrhea (72 h or more after hospitalization), and positive for glutamate dehydrogenase (GDH) [12]. Nine samples from patients with recurrent CDIs that were seeking fecal microbiota transplantation treatment [50] were also included: these samples were collected from patients from other hospitals located in São Paulo (n = 1), Rio de Janeiro (n = 1), and Minas Gerais (n = 7). Additional details regarding these samples are provided in S1 File. All stool samples were obtained in sterile containers, and aliquots were held at -20°C until all tests were performed.

#### Animals

A total of 544 samples from animals were submitted to *C*. *difficile* isolation, including 282 samples from domestic animals (canines, felines, equines, swine, and calves) and 271 samples from wild or synanthropic animals (Table 2). Most samples were subjected to differential diagnosis of enteropathogens on the diagnostic routine of the Laboratory of Anaerobic Bacteria from the Veterinary University of Minas Gerais. Other samples were obtained from three parallel studies evaluating zoonotic pathogens in pigeons (data not published), tortoises [51], and rodents and marsupials [52]. Samples from free-living rodents were obtained from two urban parks in Belo Horizonte, Minas Gerais [52]. Samples from pigeons were obtained between January 2019 and February 2020 by non-probabilistic sampling of urban pigeons (*Columbia livia* f. *urbana*) at the Veterinary Hospital of Federal University of Minas Gerais (UFMG), Brazil. Fresh feces from individual cages where the pigeons were left to rest between capture and manipulation were collected immediately after they were dropped into sterile microtubes using sterile spatulas [53]. All samples were stored at -20°C and processed within 72 h of collection. Samples from the manned wolf (*Chrysocyon brachyurus*), lions (*Panthera leo*), and jaguar (*Panthera onca*) were collected after the animals were chemically restrained by the Belo Horizonte Zoo veterinarians. Samples from all other wild animals (S1 File) were collected by the veterinary staff of the Wild Animals Screening Center (CETAS) in Belo Horizonte city (Minas Gerais, Brazil), an agency responsible for receiving, rehabilitating, and reintroducing wild animals into their natural environment.

**Table 2:** Animal species samples (n = 544) included for *Clostridioides* (*Clostridium*) *difficile* detection between February 2018 and January 2020 at the laboratory of anaerobic bacteria from the veterinary university of Minas Gerais.

Animals		Samples	*C*. *difficile* isolation		Ribotypes^1^ (Number of isolates)
			Non-toxigenic	Toxigenic	
**Domestic species (n = 282)**	Dogs	181	5	7	014(3), 106(3), 600(1)
	Horses and foals	47	6	12	078(1), 126(11)
	Cats	24	0	2	106(2)
	Piglets	19	4	13	002(1), 078(1), 126(9), 127(1), 695(1)
	Calves	11	0	0	None
**Wild and synanthropic species (n = 271)**	Rodents^2^	144	1	1	064(1)
	Tortoise (*Chelonoidis carbonaria*)	65	0	0	None
	Opossum (*Didelphis* sp.)	18	0	0	None
	Pigeons (*Columbia livia*)	18	1	0	None
	Gray Slender Opossum (*Marmosops incanus*)	7	0	0	None

#### *Clostridioides difficile* isolation and A/B toxin detection

*C*. *difficile* was isolated from fecal samples from humans and animals using a direct inoculation in cycloserine-cefoxitin-fructose agar with sodium taurocholate (TCCFA) after ethanol shock, as previously described [54]. Colonies with suggestive morphology (flat, irregular, and with ground-glass appearance) were subjected to a previously described multiplex PCR [29] and PCR ribotyping [30]. RTs not identified in the Brazilian or Leiden (Netherlands) *C*. *difficile* library were sent to the National Reference Laboratory for *C*. *difficile* (University of Leeds, United Kingdom), and a new type number was assigned when necessary. In addition, toxigenic *C*. *difficile* strains, positive for binary toxin-encoding gene (*cdtB*) and identified as new RTs, were also submitted to MLST, as previously described [55]. Stool samples positive for toxigenic *C*. *difficile* were also subjected to toxin A/B detection using an enzyme-linked immunosorbent assay kit (Ridascreen *C*. *difficile* Toxin A/B®, R-Biopharm, Germany).

### Statistical analysis

Statistical analyses for A/B toxin production, swimming motility, and spore production were performed using R Core Team (2020), and comparisons between groups were performed considering strains and time. One-way analysis of variance with post-hoc analysis by Tukey’s test was used. The results were expressed as the mean ± standard error of the mean, and all data were converted to a base 10 logarithm. To evaluate the survival analysis in hamsters, a Kaplan-Meier survival chart was constructed and analyzed using the Mantel-Cox test (GraphPad Prism 6, USA). Differences were considered significant at P <0.05.

## Results

### TcdA/TcdB and spore production and swimming motility

**Fig 1:**
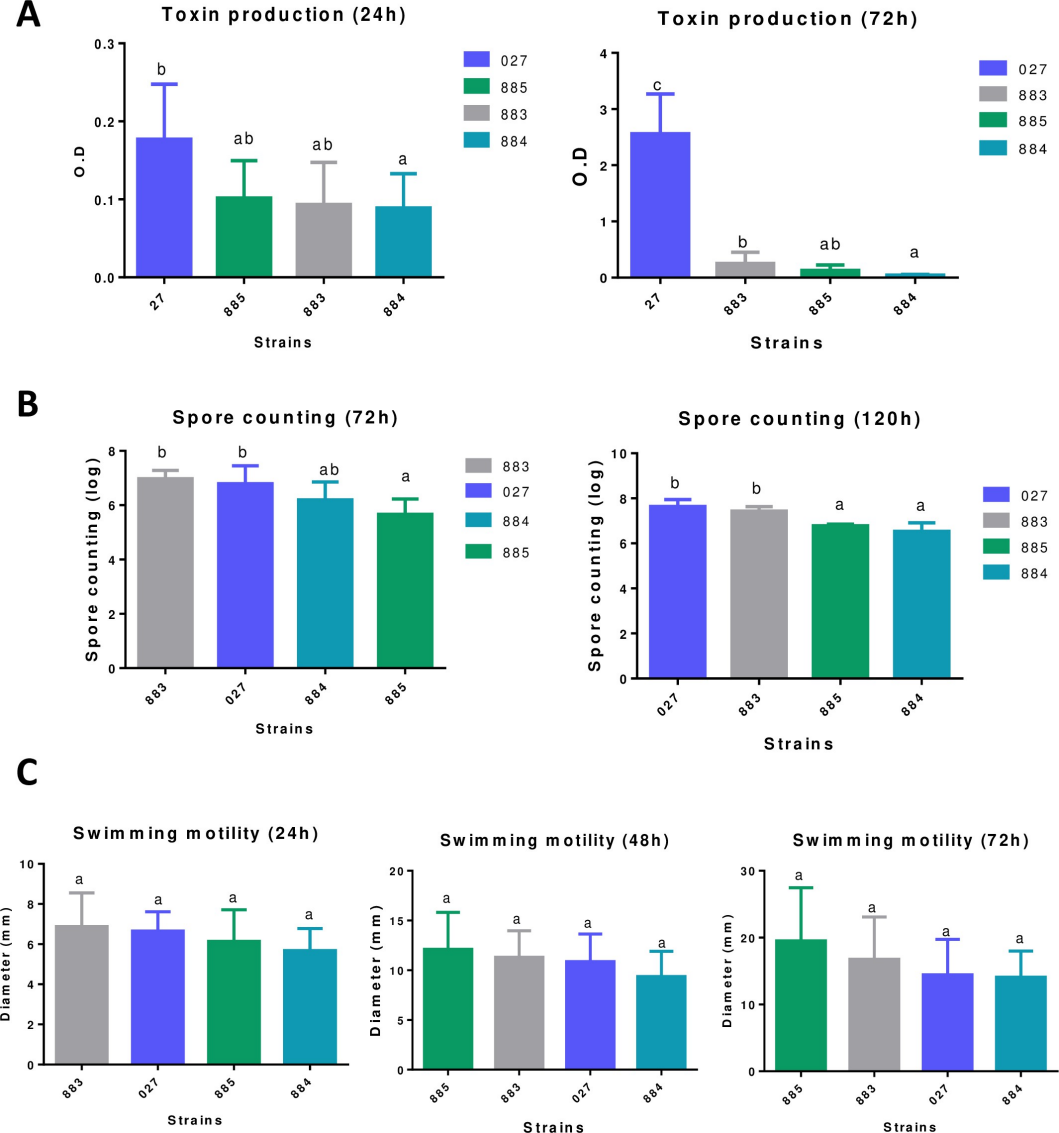
(A) A/B toxin production was measured at two different time-points using the Ridascreen *Clostridioides difficile* Toxin A/B® (R-Biopharm, Germany). (B) *Clostridioides difficile* spore counting on the log10 scale at the experimental time-points: 72 h and 120 h. (C) Diameter of the bacterial lawn of RT883, RT884, RT885, and RT027 at 24, 48, and 72 h. Data are reported as the means ± SD from three independent experiments.

At 24 h, the amount of A/B toxin produced by RT883 and 885 was similar to that of RT027; however, at 72 h, the A/B production in RT027 was substantially higher than that in all other strains (Fig 1). The spore production at 72 h ranged from 5.64 to 6.97 log10 optical density (OD), and RT883 and 027 showed similar sporulation rates (Fig 1). At 120 h, the sporulation rates ranged from 6.53 to 7.66 log10 CFU/mL, with RT883 and 027 producing similar amounts of spores.

### Antimicrobial susceptibility

The MIC values for clindamycin ranged from 2 to 8 μg/mL. RT885 was classified as antimicrobial resistant for clindamycin, and RT883 and RT884 were classified as susceptible and intermediately susceptible, respectively. All strains were susceptible to all other antimicrobials tested, including metronidazole and vancomycin.

### Lethality of all tested RTs was similar to that of RT027

**Fig 2:**
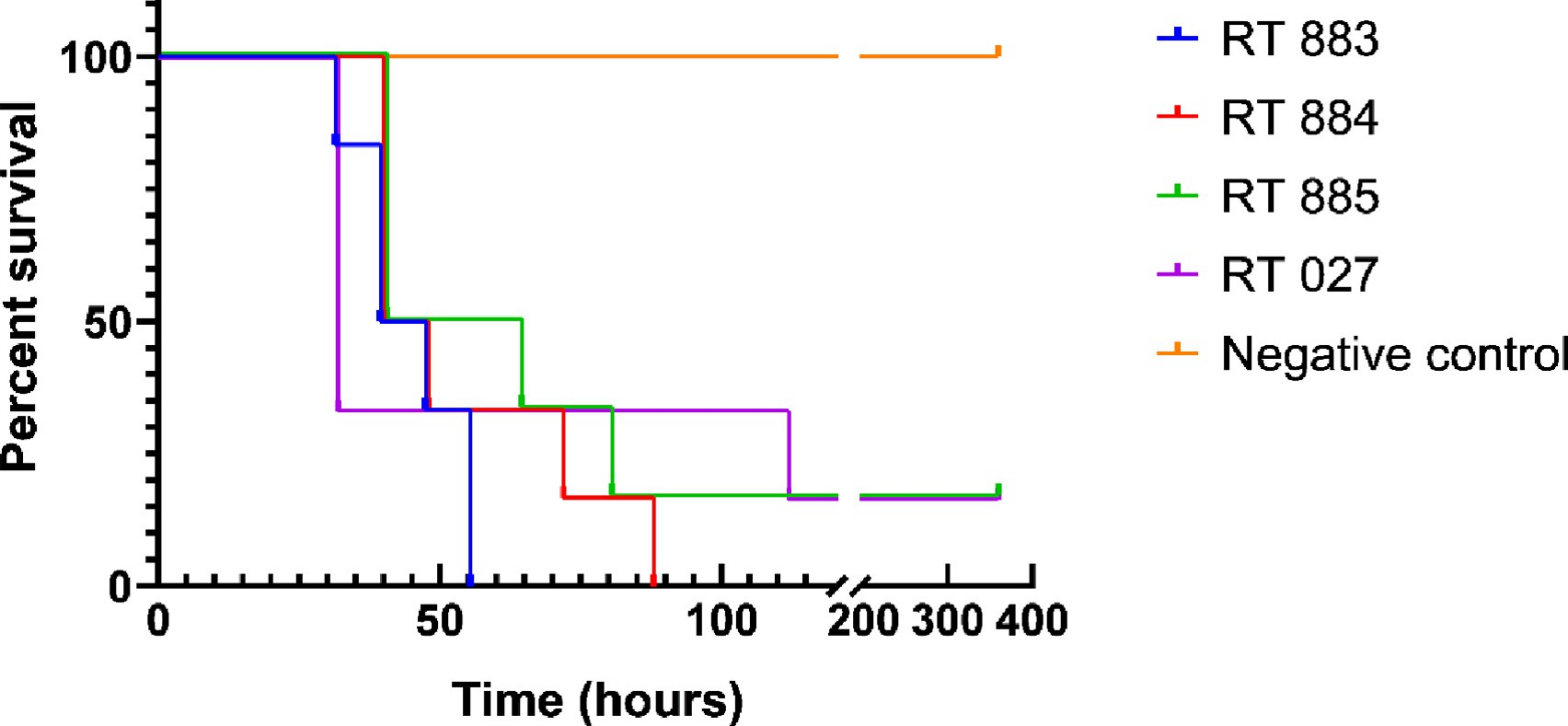
Kaplan‑Meier survival curve of hamsters infected with 10^4^ spores of RT883, RT884, RT885, reference control strain (CD196/RT027), and negative control. Syrian golden hamsters previously treated with clindamycin were orally inoculated with spores from the indicated strains. Hamsters were monitored at 8-h intervals for signs of *Clostridioides difficile* infection for 15 days, and the number of deceased animals was recorded.

All strains tested were capable of causing diarrhea and death in the animals (Fig 2). In addition, all animals that died during the experiment were positive for the isolation of *C*. *difficile* and its toxins. No macroscopic lesions were seen in all animals from the negative control group and in two surviving animals from groups I and III (inoculated with RT027 and RT885, respectively). These animals were also negative for *C*. *difficile* and A/B toxins in the intestinal content at day 15. In the *post-mortem* examination, the mucosa and serosa of the cecum of the animals that died were extensively hemorrhagic and had a bloody appearance (S2 File). There was no significant difference in animal survival between the groups (I to V). Thus, all tested RTs showed lethality statistically similar to the control group (I) challenged with the reference strain RT027.

### Comparative genomics

**Fig 3:**
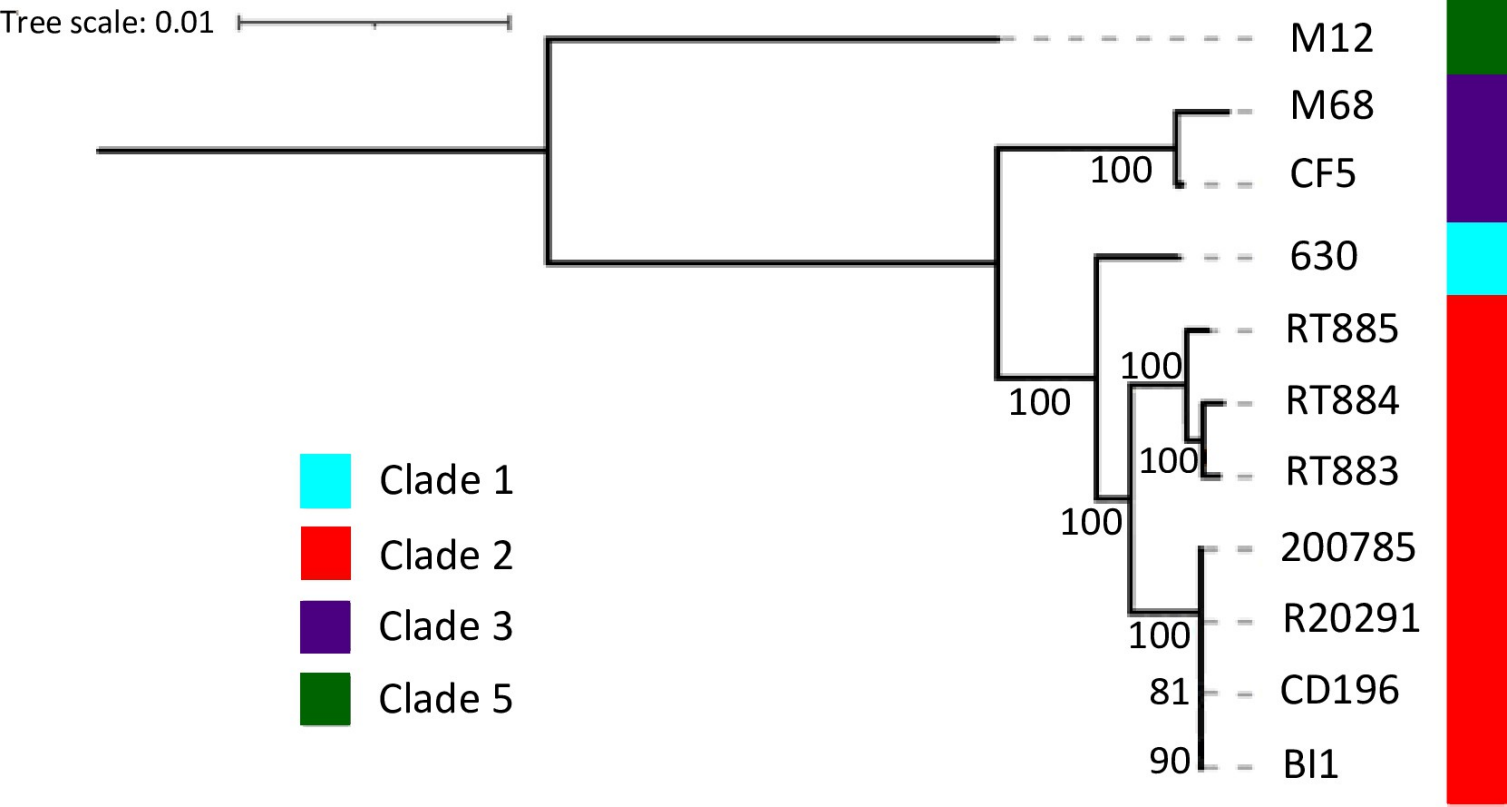
cgMLSA maximum likelihood phylogeny. The cgMLSA phylogeny tree based in 2.643 genes, containing 2,531,373 positions rooted by midpoint is represented. The colour strip represents the *C*. *difficile* Clades, where Clade 1, 2, 3 and 5 are represented by respectively the colours cyan, red, purple and green. The values in the branches correspond to the percentage of support (0 to 100%) based on 1000 ultrafast bootstrap replicates. The figure represents that RT883, 884, and 885 are very similar to each other and are related to other clade 2 reference strains (R20291, CD196, BI1, 2007855). Reference strains from clade 1 (CD630), clade 3 (M68, CF5), and clade 5 (M120) were also added for comparison purpose.

Both cgMLST and cgMLSA analyses showed that the three evaluated strains clustered in a single clade, that emerged from a common ancestor from clade 2 (Fig 3). However, the long branch separating these three isolated from other clade2 strains and their ANI of ~99.5% (S2 File) suggests that they may have diverged a long time ago.

**Fig 4:**
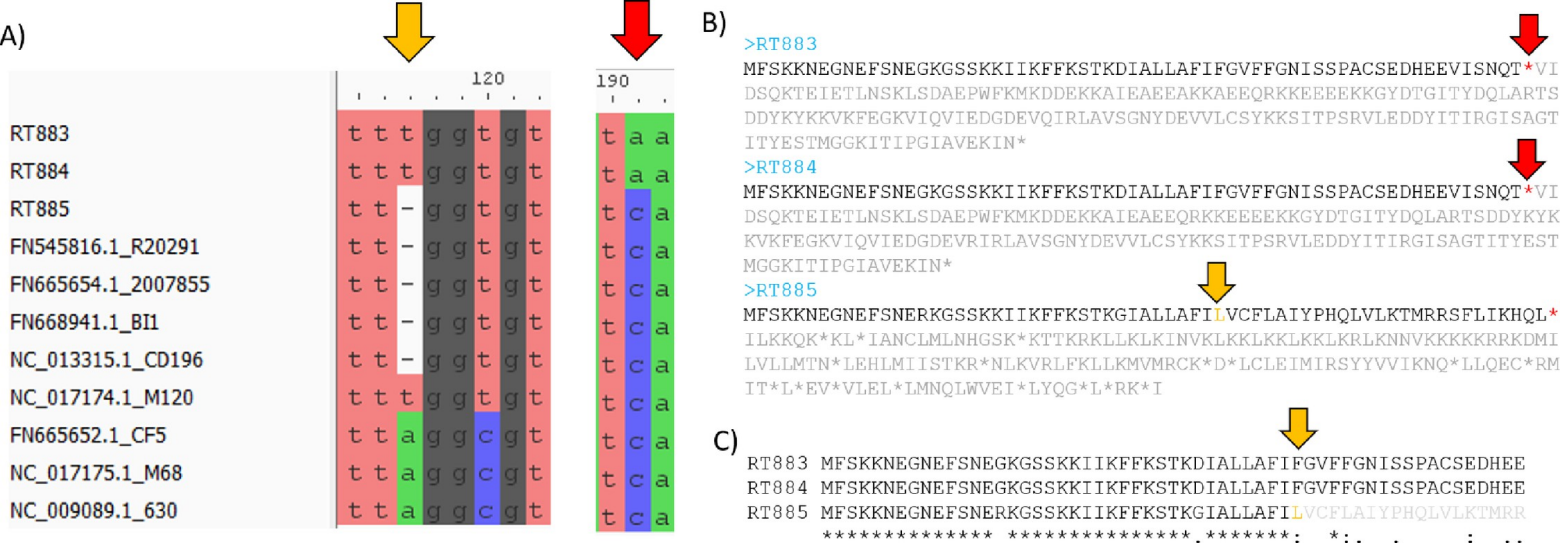
*Clostridioides difficile* PaLoc *tcdC* gene structure. (A) A Partial multiple alignment of the *tcdC* gene. The C→A mutation of the RT883 and 884 isolates at position 191 that generates a “TAA” stop codon is highlighted by a red arrow, while the point deletion that resulted in a frameshift in 885 is highlighted by an orange arrow. (C) Amino acid sequences encoded by the *tcdC* genes in RT883, 884, and 885 are shown; the stop codons are represented by red asterisks. Positions after the first stop codons are represented in gray. C) Multiple alignment of the *tcdC* gene in RT883, RT884 and RT885. Asterisks at the bottom denotes conserved sequences in all isolates. The orange arrow represents the frameshift position in RT885.

**Fig 5:**
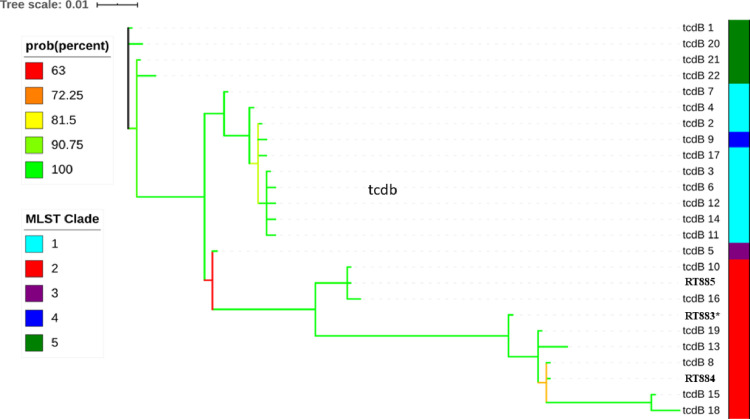
Phylogenetic analysis by Bayesian estimation of the amino acid sequences of *tcdB* alleles from the three Brazilian clade 2 strains (RT883, 884, and 885). The distribution of alleles according to different clades is shown on color strip in the right. Posterior probability branch support is denoted by a color code, from red (65% support) to green (100% support). (*) A novel allele (*tcdB* 23) was identified in RT883, while *tcdB* alleles 8 and 10 were identified in RT884 and RT885, respectively.

Analysis of the PaLoc structure demonstrated that RT883 and RT884 contain an internal stop codon on *tcdC* genes, while in RT885, *tcdC* has a reading frameshift (Fig 4). New *tcdB* and *tcdC* alleles were identified and deposited in the PubMLST database as allele numbers 23 and 57, respectively (S3 File). The TcdB amino acid sequences identified in the three Brazilian strains were similar to those of other sequences from clade 2 strains (Fig 5).

The *ermB* and *tetM* genes, commonly associated with macrolides and tetracycline resistance [56], were not detected. Typical fluoroquinolone resistance mutations were not identified in the *gyrA* and *gyrB* alleles (S3 File). Analysis of specific regions revealed structures in Brazilian isolates that were potentially derived from prophages and transposons. Notably, the matches were only partial, approximately 1 kb, and the transposons were approximately 10 kb in size; the only exception was HG475346 (in green), which presented larger matches (S3 File). In addition, several virulence-related genes such as adhesins and flagellins were detected in these strains by in silico PCR (S3 File).

### Isolation and ribotyping of *C*. *d*ifficile from humans and animals (2018 to 2020)

**Fig 6:**
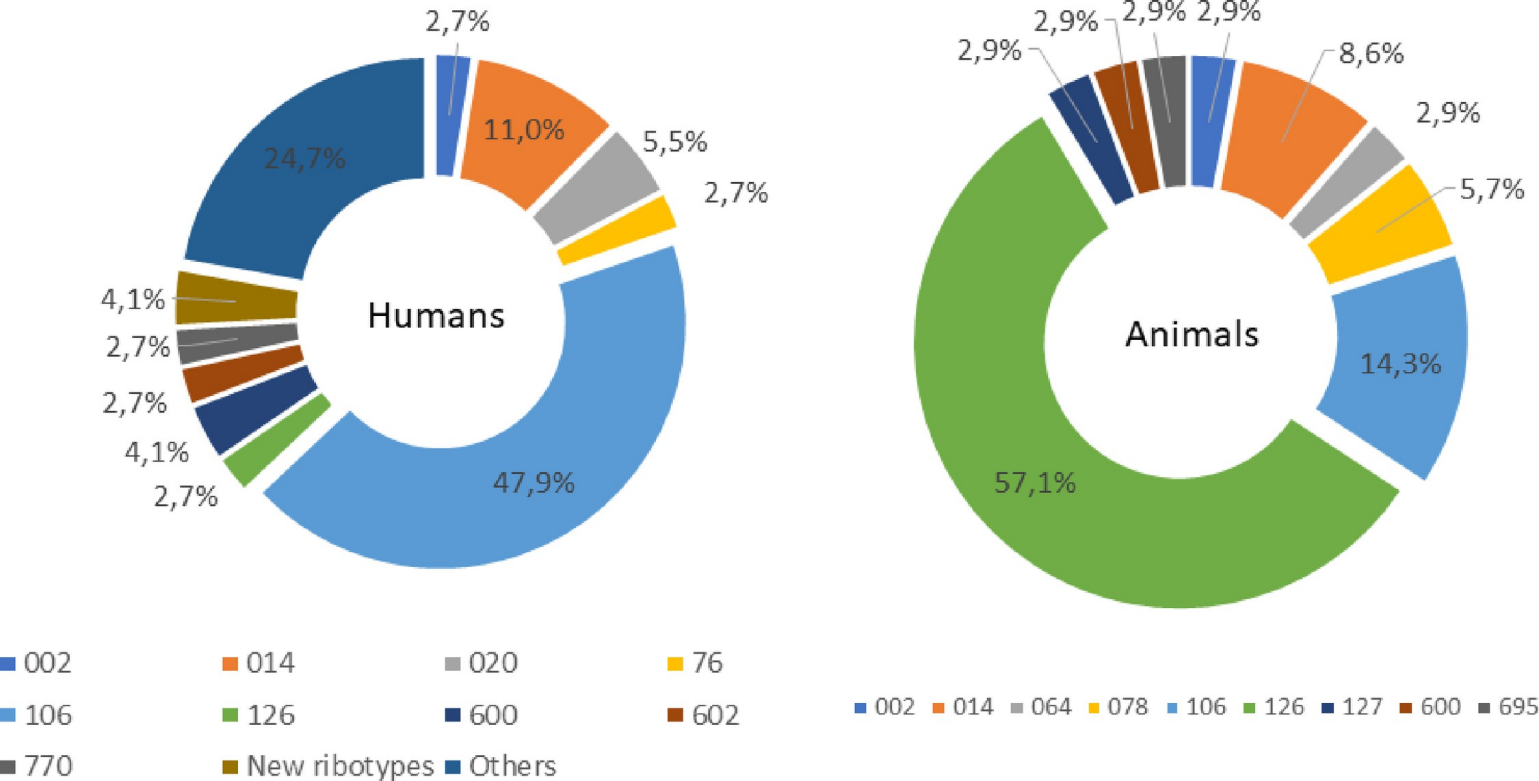
Distribution of *Clostridioides difficile* ribotypes from toxigenic strains isolated from humans (n = 86) and animals (n = 35) in Brazil between 2018 and 2020.

A total of 125 *C*. *difficile* isolates were obtained from 152 diarrheic human samples (82.2%). Of these isolates, 39 (31.2%) were non-toxigenic and 86 (68.8%) were toxigenic (all A+B+). Among toxigenic isolates, a total of 30 different RTs were detected (S3 File). RT883, 884, and 885, as well as the classic epidemic RT027 and 078, were not isolated. RT126 was detected in two patients. RT106 and 014/020 were the most frequent RTs in human samples (47.9% and 11%, respectively) (Fig 6). Three new RTs were identified: RT988, 989, and 990.

A total of 55 (10.1%) *C*. *difficile* strains were isolated from 543 animal samples tested. Among the isolates, 20 (36.4%) were non-toxigenic. Among the 35 toxigenic isolates, nine RTs were detected, with RT126 and 014/020 being the most frequent (59.4% and 9.4%, respectively) (Fig 6). Similar to the results obtained for human samples, RT027 was not detected while RT078 was isolated from two animals (6.2%): one foal and one piglet.

**Fig 7:**
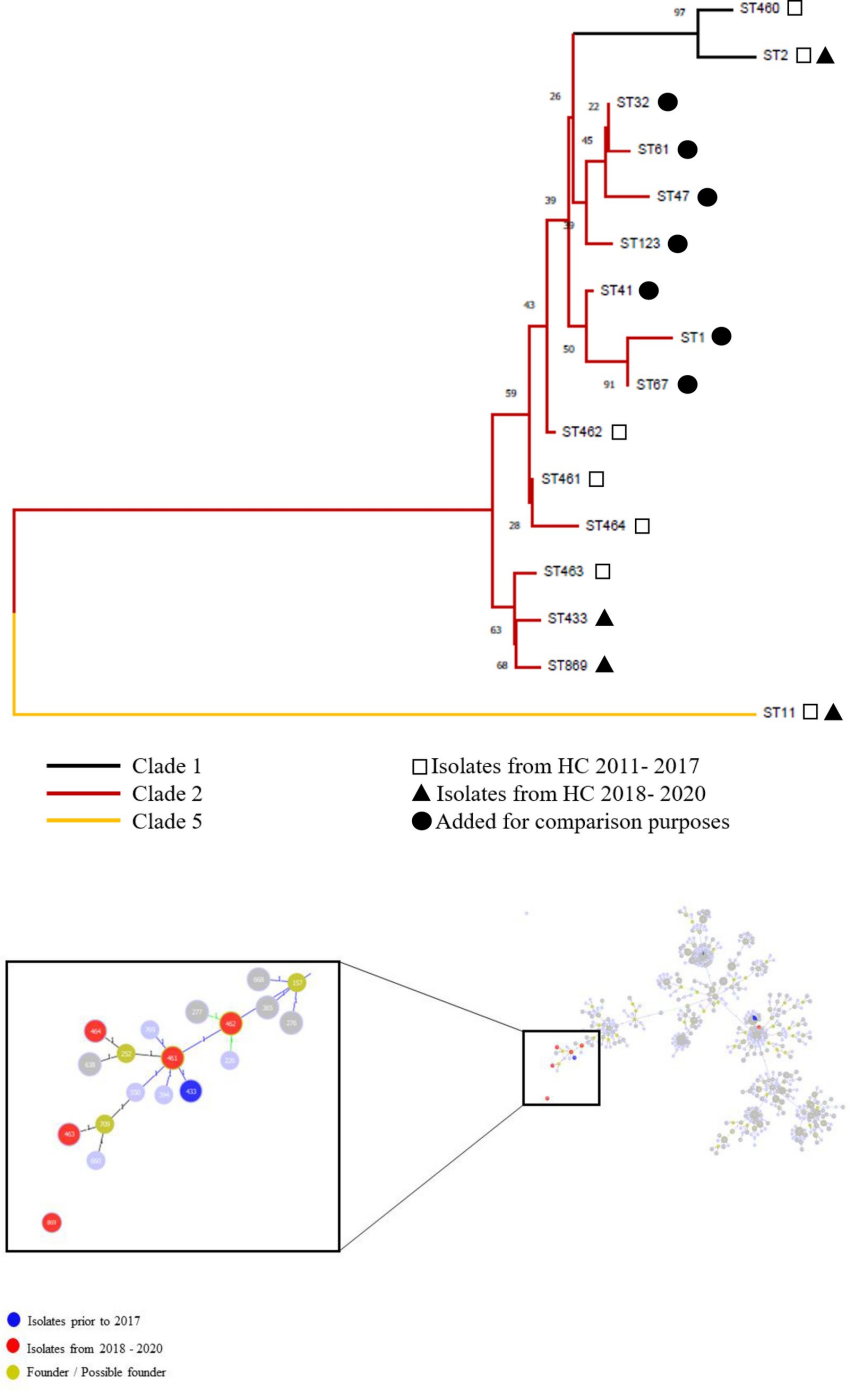
Neighbour-joining tree generated from concatenated sequences of the seven housekeeping genes of all the 16 STs identified in the present study. The novel STs are marked with asterisks (*). ST1, ST2, ST11, ST32, ST47, ST123, ST41, and ST67 (●) were added for comparison purposes. Software: The Molecular Evolution and Genetic Analysis 7.0.26 (MEGA7). B) eBURST analysis of *Clostridioides difficile* using STs available in the MLST database in December 2021. Each ST is represented by a circle, and lines connect single-locus variants. Khaki circles represent predicted founders. Detail showing the relation of four out of five STs from clade 2 detected in the present study (black arrows), including three Brazilian STs (462, 463, and 464). The remaining new sequence type (ST869) was a singleton.

Two of the three novel RTs isolated from humans (RT988 and 989) were positive for the binary toxin-encoding gene (*cdtB*) and were then submitted to MLST. RT989 was classified as ST433, whereas RT988 was classified as ST869, a new ST, both from clade 2. In order to compare these two new isolates (RT988 and 989) with RT883, 884, 885, a neighbor-joining tree was constructed using concatenated MLST sequences. This analysis revealed that Brazilian clade 2 strains are more similar to each other than to the classic strains from clade 2, including RT027 (ST1 and ST67) and RT244 (ST41). Interestingly, this similarity seems to be even higher between the two strains isolated recently (2018 to 2020) and one of the strains isolated in 2015 and evaluated in the present study (RT885/ST463) (Fig 7). An eBURST analysis performed in December 2021 using *C*. *difficile* STs available in the MLST database also showed that four of these Brazilian clade 2 isolates grouped together (RT883/ST461, RT884/ST462, RT424/ST464, RT885/ST463, and RT989/ST433), whereas RT988/ST869 was a singleton (Fig 7).

## Discussion

Epidemiological data on CDI in the Brazilian territory are available [13]. To date, epidemic RTs commonly associated with outbreaks and severe cases, such as RT027 and RT078, are extremely rare in Brazil in both humans and animals [8–10, 12, 16, 54]; thus, the epidemiology of CDI in Brazil strongly differs from that of North America and most European countries. In addition, novel strains of clade 2, the clade associated with outbreaks in different regions [4, 57, 58], have been reported in hospitals in Brazil [9, 15, 16]. These findings raised the hypothesis that possible novel epidemic strains could be circulating in Brazil, as has also reported in some European countries as Greece (RT181) and Czech Republic (RT176) [17, 59]. Thus, in order to better understand the possible circulation of these strains and the risk it might represent, the present study assessed the phenotypic and genetic characteristics of three new Brazilian ST/RT strains from clade 2. In addition, a two-year survey was conducted in animals and humans to verify the circulation of these strains as well as other possible epidemic RTs.

In the present study, all strains were phenotypically susceptible to moxifloxacin, and no amino acid substitutions known to be associated with resistance were identified in *gyrA* or *gyrB*. These results are relevant because the resistance to fluoroquinolones strongly contributed to the spread of *C*. *difficile* RT027 and other *C*. *difficile* strains, which later were associated with outbreaks and increased morbidity and mortality due to CDI [57, 60]. In addition, all *C*. *difficile* isolates in the present study were susceptible to metronidazole and vancomycin, the drugs of choice for treating CDI in humans [61–63].

In all three strains evaluated, the function of the *tcdC* gene was affected by an internal stop codon (RT883 and RT884) or by a reading frameshift (RT885) (Fig 4), similar to that previously reported in epidemic RT027 isolates [2]. Although some studies have suggested that deletions in the negative regulator *tcdC* result in increased toxin production [63–66], further studies have refuted this association, suggesting that the deletion of the *tcdC* gene may not influence the epidemic potential of an isolate [66–69]. In fact, the toxin production of all three strains tested was inferior to that of RT027 (Fig 1). According to Stabler et al. [66], although toxin production level is a relevant characteristic, the combination of several factors, such as antibiotic resistance and increased transmissibility manifested through sporulation and motility, might explain the emergence of RT027 as well as other epidemic strains.

TcdB is considered as an important virulence factor in CDI. In this study, a novel allele was identified in one of the strains, and all three sequences of the TcdB-encoding gene showed high similarity with previously reported sequences of *tcdB* from other clade 2 strains (Fig 5). This finding is interesting because TcdB subtypes do not completely align with the phylogeny of *C*. *difficile* [70]. In fact, of the two major toxins of CDI, TcdB is known to have a higher variation owing to accelerated evolution [70]. Furthermore, amino acid differences in TcdB can impact CDI pathogenesis. Recently, Ramírez-Vargas [26] showed that different *tcdB* alleles could induce different cytopathic effects, indicating distinct levels of cytotoxicity in cell culture, leading to differences in the lethality rate in the hamster model. In addition, different alleles could yield a different TcdB structure and alter the performance of laboratory diagnostic tests and even treatment based on antitoxins [26, 70]. Further studies are needed to evaluate whether the amino acid alterations seen in this novel TcdB would somehow affect its biological activity.

All tested strains, including the control RT027, caused death at a similar rate and proportion in the hamster model (Fig 5). This result was surprising, as TcdA and TcdB are known as the major causes of CDI symptoms, and all three tested strains showed a lower toxin production than the reference RT027 [26, 71, 72]. Few studies have examined the in vivo virulence of different RTs using animal models, but hamsters have been successfully used in these survival studies because of their high sensitivity to CDI [26, 73–75]. Previous reports have shown that isolates of epidemic RTs were found to be more virulent in hamster models than non-epidemic isolates, but the in vitro profiles of individual isolates were not always predictive of their in vivo virulence, similar to our findings [75, 76]. According to Vitucci et al. [76], toxin levels may represent one of the relevant factors involved in disease severity. In fact, *C*. *difficile* strains with higher colonization capability can cause severe infection even with low toxin production [19]; these findings can partially explain the results observed in the present study.

Furthermore, all tested strains showed motility similar to that of RT027 (Fig 1). Flagella are important for motility in several enteric pathogens, contributing to the adherence and colonization in gut epithelial cells [19, 66]. In addition, the spore count of one strain (RT883) was similar to that of RT027 at 120 h (Fig 3). Similar to motility, spore formation is also linked to the pathogenesis of CDI and is an important factor for transmission and persistence of the pathogen, improving the presence and spread of *C*. *difficile* in the population [77]; additionally, it is directly linked to disease relapse [78]. Studies have suggested that sporulation rates are high not only in epidemic *C*. *difficile* strains but also in worldwide prevalent endemic strains, including RT014/020, reinforcing that sporulation is a relevant factor in the dissemination of *C*. *difficile* [21, 77].

The cgMLST and cgMLSA revealed a high degree of similarity among the isolates, with ANI ~99.5%, suggesting an evolution from a common ancestor. Moreover, these strains share a common ancestor with the classic clade 2 strains, including RT027 (ST1 and ST67) and RT244 (ST41). However, these Brazilian isolates form an individual clade separated from the clade containing classic group 2 strains, suggesting a recent divergence (Fig 3). Notably, this finding is in accordance with Stabler et al. [69], who suggested the presence of divergent subclades within clade 2 strains, indicating that these lineages may evolve rapidly and diverge into potentially new virulent clones.

To verify whether these three novel clade 2 strains were still circulating and to better understand the presence of other potential epidemic strains, we conducted a two-year survey in the same hospital where these strains were first isolated. Samples from patients from other hospitals who underwent fecal microbiota transplantation at our institution were also included. In addition to the human samples, several animals were tested for *C*. *difficile* during this period. A total of 139 *C*. *difficile* isolates were obtained between 2018 and 2020, and ribotyping of the toxigenic strains did not reveal the presence of RT883, RT884, or RT885. In fact, more than half of the strains isolated from humans were RT106 and RT014/020, which were also detected in dogs and cats during this period. These results are similar to those of other studies in Brazil, suggesting that the two aforementioned strains are the most common RTs and are also frequently isolated from humans and animals [13, 16, 54]. Interestingly, in recent years, several reports have suggested a progressive increase in the incidence of CDI caused by RT106 in several countries [79–81]. At our institution, the frequency of RT106 increased from 20.6% in a study conducted between 2011 and 2015 [12] to 47.9% in the present study, conducted from 2018 to 2020; these data suggest the need for further studies focusing on this specific RT.

RT027 was not detected in samples from both animals and humans in Brazil. So far, it has been reported only in two individuals in 2018 in Southern Brazil [14]. However, typical clade 5 strains (RT078 and RT126) were detected in two patients (2.7%) but corresponded to more than 60% of the animal isolates in the present study. These results corroborate those of previous studies that failed to detect RT078 in humans in Brazil, whereas both RT078 and 126 have been already reported in several animal species, including swine, horses, and rodents [8, 10, 13, 82, 83]. This finding contrasts with reports in European countries and in North America, where RT078 seems to be common in both humans and animals [84, 85]. Notably, although RT078 can cause healthcare-associated CDI, it has been associated with community-acquired cases [86]. Owing to the high genetic similarity of the RT078 strains isolated from humans and animals, a possible zoonotic and/or anthroponotic transmission between animals and humans with contaminated food and environment acting as a conduit between the two has been suggested [84, 87]. This study focused on healthcare-associated CDI, and the absence of samples from patients with community-acquired CDI in the present study can, at least partially, explain the absence of RT078. Unfortunately, there are no studies related to community-acquired CDI in Brazil, and therefore, the epidemiology of these cases is still unclear.

New RTs were not isolated from animals, whereas in humans, three novel strains (RT988, 989, and 990) were identified. Two of these new RTs were *cdtB*-positive and belonged to clade 2. A neighbor-joining tree based on the concatenated sequences from MLST suggests that these two new RTs (RT989/ST433 and RT988/ST869) are similar to those strains characterized in the present study (RT885/ST463). Moreover, these strains are located in a different branch from RT027/ST1, ST67, and RT244/ST41, supporting the idea that at least two subclades are evolving within clade 2 [69]. Similarly, eBURST analysis revealed that the genetic similarities between each Brazilian clade 2 isolate were higher than those between the Brazilian clade 2 isolates and the classic hypervirulent strains. This finding suggests that novel clade 2 strains, which are highly similar to each other, continue to emerge in our setting, indicating the need for long-term local surveillance in order to identify lineages that can become endemic or epidemic, as seen in other countries [11, 80]. Interestingly, one of these novel strains (RT885/ST463) was recently detected in a patient with severe CDI in another institution in São Paulo [16]; this finding confirms that these novel clade 2 strains are circulating in Brazil.

Further understanding of global CDI epidemiology is hindered by a lack of surveillance, especially in the developing world [88]. In this context, the present work contributes to the literature by evaluating three novel clade 2 strains and conducting a large-scale characterization of *C*. *difficile* RTs circulating in humans and animals in Brazil. Based on the in vivo and in vitro results, the three novel clade 2 strains studied showed some potential for becoming endemic or epidemic. In addition, the present study has revealed the circulation of two new clade 2 RTs, confirming that these strains continue to emerge in our setting.

## Supporting information

S1 File Details of the animals and humans stool samples included in the present study. (XLSX)

S2 File Fast ANI results. (XLSX)

S3 File Supplementary tables and figures. (PDF)

## References

[1] Barbanti F, Spigaglia P. Microbiological characteristics of human and animal isolates of Clostridioides difficile in Italy: Results of the Istituto Superiore di Sanità in the years 2006–2016. Anaerobe. 2020 Feb;61:102136. doi: 10.1016/j.anaerobe.2019.102136 31857201

[2] Warny M, Pepin J, Fang A, Killgore G, Thompson A, Brazier J, et al. Toxin production by an emerging strain of Clostridium difficile associated with outbreaks of severe disease in North America and Europe. The Lancet. 2005 Sep;366(9491):1079–84. doi: 10.1016/S0140-6736(05)67420-X 16182895

[3] Pituch H, Obuch-Woszczatyński P, Lachowicz D, Wultańska D, Karpiński P, Młynarczyk G, et al. Hospital-based Clostridium difficile infection surveillance reveals high proportions of PCR ribotypes 027 and 176 in different areas of Poland, 2011 to 2013. Eurosurveillance. 2015 Sep 24;20(38). 10.2807/1560-7917.ES.2015.20.38.30025 26536049

[4] Hong S, Knight DR, Chang B, Carman RJ, Riley T v. Phenotypic characterisation of Clostridium difficile PCR ribotype 251, an emerging multi-locus sequence type clade 2 strain in Australia. Anaerobe. 2019 Dec;60:102066. doi: 10.1016/j.anaerobe.2019.06.019 31260740

[5] Lim SK, Stuart RL, Mackin KE, Carter GP, Kotsanas D, Francis MJ, et al. Emergence of a Ribotype 244 Strain of Clostridium difficile Associated With Severe Disease and Related to the Epidemic Ribotype 027 Strain. Clinical Infectious Diseases. 2014 Jun 15;58(12):1723–30. doi: 10.1093/cid/ciu203 24704722

[6] Davies KA, Ashwin H, Longshaw CM, Burns DA, Davis GL, Wilcox MH. Diversity of Clostridium difficile PCR ribotypes in Europe: results from the European, multicentre, prospective, biannual, point-prevalence study of Clostridium difficile infection in hospitalised patients with diarrhoea (EUCLID), 2012 and 2013. Eurosurveillance. 2016 Jul 21;21(29). doi: 10.2807/1560-7917.ES.2016.21.29.30294 27470194

[7] Eyre DW, Tracey L, Elliott B, Slimings C, Huntington PG, Stuart RL, et al. Emergence and spread of predominantly community-onset Clostridium difficile PCR ribotype 244 infection in Australia, 2010 to 2012. Eurosurveillance. 2015 Mar 12;20(10). 10.2807/1560-7917.es2015.20.10.21059 25788254

[8] de Almeida Monteiro A, Pires RN, Persson S, Filho EMR, Pasqualotto AC. A search for Clostridium difficile ribotypes 027 and 078 in Brazil. The Brazilian Journal of Infectious Diseases. 2014 Nov;18(6):672–4. doi: 10.1016/j.bjid.2014.08.004 25307680 PMC9425211

[9] Diniz AN, de Oliveira Júnior CA, Vilela EG, Figueiredo HCP, Rupnik M, Wilcox MH, et al. Molecular epidemiology of Clostridioides (previously Clostridium) difficile isolates from a university hospital in Minas Gerais, Brazil. Anaerobe. 2019 Apr;56:34–9. doi: 10.1016/j.anaerobe.2019.01.010 30703440

[10] Silva ROS, Rupnik M, Diniz AN, Vilela EG, Lobato FCF. Clostridium difficile ribotypes in humans and animals in Brazil. Memórias do Instituto Oswaldo Cruz. 2015 Dec 11;110(8):1062–5. doi: 10.1590/0074-02760150294 26676318 PMC4708028

[11] Li C, Liu S, Zhou P, Duan J, Dou Q, Zhang R, et al. Emergence of a Novel Binary Toxin–Positive Strain of *Clostridium difficile* Associated With Severe Diarrhea That Was Not Ribotype 027 and 078 in China. Infection Control & Hospital Epidemiology. 2015 Sep 11;36(9):1112–4. 26062650 10.1017/ice.2015.120

[12] Lopes Cançado GG, Silveira Silva RO, Rupnik M, Nader AP, Starling de Carvalho J, Miana de Mattos Paixão G, et al. Clinical epidemiology of Clostridium difficile infection among hospitalized patients with antibiotic-associated diarrhea in a university hospital of Brazil. Anaerobe. 2018 Dec;54:65–71. doi: 10.1016/j.anaerobe.2018.08.005 30114442

[13] Trindade CNR, Domingues RMCP, Ferreira EO. The epidemiology of Clostridioides difficile infection in Brazil: A systematic review covering thirty years. Anaerobe. 2019 Aug;58:13–21. doi: 10.1016/j.anaerobe.2019.03.002 30851427

[14] Pires RN, Monteiro AA, Saldanha GZ, Falci DR, Caurio CFB, Sukiennik TCT, et al. Hypervirulent *Clostridium difficile* Strain Has Arrived in Brazil. Infection Control & Hospital Epidemiology. 2018 Mar 25;39(3):371–3. 29368667 10.1017/ice.2017.280

[15] Costa CL, López-Ureña D, de Oliveira Assis T, Ribeiro RA, Silva ROS, Rupnik M, et al. A MLST Clade 2 Clostridium difficile strain with a variant TcdB induces severe inflammatory and oxidative response associated with mucosal disruption. Anaerobe. 2016 Aug;40:76–84. doi: 10.1016/j.anaerobe.2016.06.005 27311833

[16] Girão ES, de Melo Tavares B, dos Santos SA, Gamarra GL, Rizek C, Martins RC, et al. Predictive factors, outcomes, and molecular epidemiology of Clostridioides difficile diarrhea in Brazilian hospitals. European Journal of Clinical Microbiology & Infectious Diseases. 2021 Aug;40(9):1821–32. 33783664 10.1007/s10096-021-04189-3

[17] Kachrimanidou M, Baktash A, Metallidis S, Tsachouridou Ο, Netsika F, Dimoglou D, et al. An outbreak of Clostridioides difficile infections due to a 027-like PCR ribotype 181 in a rehabilitation centre: Epidemiological and microbiological characteristics. Anaerobe. 2020 Aug;65:102252. doi: 10.1016/j.anaerobe.2020.102252 32781108

[18] McDonald LC, Gerding DN, Johnson S, Bakken JS, Carroll KC, Coffin SE, et al. Clinical Practice Guidelines for Clostridium difficile Infection in Adults and Children: 2017 Update by the Infectious Diseases Society of America (IDSA) and Society for Healthcare Epidemiology of America (SHEA). Clinical Infectious Diseases. 2018 Aug;66(7):987–94. doi: 10.1093/cid/ciy149 29562266

[19] Li C, Harmanus C, Zhu D, Meng X, Wang S, Duan J, et al. Characterization of the virulence of a non-RT027, non-RT078 and binary toxin-positive *Clostridium difficile* strain associated with severe diarrhea. Emerging Microbes & Infections. 2018 Dec 1;7(1):1–11. 30542069 10.1038/s41426-018-0211-1 PMC6291415

[20] Silva ROS, Guedes RMC, Gabardo M de P, Oliveira Junior CA, Salvarani FM, Pires PS, et al. Padronização de um modelo de infecção por Clostridium difficile em hamsters sírios Mesocricetus auratus. Ciência Rural. 2014;44(8):1415–21.

[21] Zidaric V, Rupnik M. Sporulation properties and antimicrobial susceptibility in endemic and rare Clostridium difficile PCR ribotypes. Anaerobe. 2016 Aug;39:183–8. doi: 10.1016/j.anaerobe.2016.04.010 27095618

[22] O’Connor JR, Galang MA, Sambol SP, Hecht DW, Vedantam G, Gerding DN, et al. Rifampin and Rifaximin Resistance in Clinical Isolates of *Clostridium difficile*. Antimicrobial Agents and Chemotherapy. 2008 Aug;52(8):2813–7. 18559647 10.1128/AAC.00342-08 PMC2493101

[23] EUCAST—European Committee on Antimicrobial Susceptibility Testing. Clinical breakpoints for bacteria. Vol. 9. 2018.

[24] Buckley AM, Spencer J, Maclellan LM, Candlish D, Irvine JJ, Douce GR. Susceptibility of Hamsters to Clostridium difficile Isolates of Differing Toxinotype. PLoS ONE. 2013 May 21;8(5):e64121. doi: 10.1371/journal.pone.0064121 23704976 PMC3660315

[25] Ramírez-Vargas G, López-Ureña D, Badilla A, Orozco-Aguilar J, Murillo T, Rojas P, et al. Novel Clade C-I Clostridium difficile strains escape diagnostic tests, differ in pathogenicity potential and carry toxins on extrachromosomal elements. Scientific Reports. 2018 Aug;8(1):13951. doi: 10.1038/s41598-018-32390-6 30224751 PMC6141592

[26] Sambol SP, Tang JK, Merrigan MM, Johnson S, Gerding DN. Infection of Hamsters with Epidemiologically Important Strains of *Clostridium difficile*. The Journal of Infectious Diseases. 2001 Jun 15;183(12):1760–6. 11372028 10.1086/320736

[27] Orozco-Aguilar J, Alfaro-Alarcón A, Acuña-Amador L, Chaves-Olarte E, Rodríguez C, Quesada-Gómez C. In vivo animal models confirm an increased virulence potential and pathogenicity of the NAP1/RT027/ST01 genotype within the Clostridium difficile MLST Clade 2. Gut Pathogens. 2020 Dec 22;12(1):45. doi: 10.1186/s13099-020-00383-4 32983262 PMC7510272

[28] Oliveira PH, Ribis JW, Garrett EM, Trzilova D, Kim A, Sekulovic O, et al. Epigenomic characterization of Clostridioides difficile finds a conserved DNA methyltransferase that mediates sporulation and pathogenesis. Nature Microbiology. 2020 Jan 25;5(1):166–80. doi: 10.1038/s41564-019-0613-4 31768029 PMC6925328

[29] Silva ROS, Salvarani FM, Cruz Júnior EC da C, Pires PS, Santos RLR, Assis RA de, et al. Detection of enterotoxin A and cytotoxin B, and isolation of Clostridium difficile in piglets in Minas Gerais, Brazil. Ciência Rural. 2011 Jul 29;41(8):1430–5.

[30] Fawley WN, Knetsch CW, MacCannell DR, Harmanus C, Du T, Mulvey MR, et al. Development and Validation of an Internationally-Standardized, High-Resolution Capillary Gel-Based Electrophoresis PCR-Ribotyping Protocol for Clostridium difficile. PLOS ONE. 2015 Feb 13;10(2):e0118150. doi: 10.1371/journal.pone.0118150 25679978 PMC4332677

[31] Bolger AM, Lohse M, Usadel B. Trimmomatic: a flexible trimmer for Illumina sequence data. Bioinformatics. 2014 Aug 1;30(15):2114–20. doi: 10.1093/bioinformatics/btu170 24695404 PMC4103590

[32] Li H, Handsaker B, Wysoker A, Fennell T, Ruan J, Homer N, et al. The Sequence Alignment/Map format and SAMtools. Bioinformatics. 2009 Aug 15;25(16):2078–9. doi: 10.1093/bioinformatics/btp352 19505943 PMC2723002

[33] Bankevich A, Nurk S, Antipov D, Gurevich AA, Dvorkin M, Kulikov AS, et al. SPAdes: A New Genome Assembly Algorithm and Its Applications to Single-Cell Sequencing. Journal of Computational Biology. 2012 May;19(5):455–77. doi: 10.1089/cmb.2012.0021 22506599 PMC3342519

[34] Galardini M, Biondi EG, Bazzicalupo M, Mengoni A. CONTIGuator: a bacterial genomes finishing tool for structural insights on draft genomes. Source Code for Biology and Medicine. 2011 Dec 21;6(1):11. doi: 10.1186/1751-0473-6-11 21693004 PMC3133546

[35] Walker BJ, Abeel T, Shea T, Priest M, Abouelliel A, Sakthikumar S, et al. Pilon: An Integrated Tool for Comprehensive Microbial Variant Detection and Genome Assembly Improvement. PLoS ONE. 2014 Aug;9(11):e112963. doi: 10.1371/journal.pone.0112963 25409509 PMC4237348

[36] Jain C, Rodriguez-R LM, Phillippy AM, Konstantinidis KT, Aluru S. High throughput ANI analysis of 90K prokaryotic genomes reveals clear species boundaries. Nature Communications. 2018 Dec 30;9(1):5114. doi: 10.1038/s41467-018-07641-9 30504855 PMC6269478

[37] Huson DH, Bryant D. Application of Phylogenetic Networks in Evolutionary Studies. Molecular Biology and Evolution. 2006 Feb 1;23(2):254–67. doi: 10.1093/molbev/msj030 16221896

[38] Alikhan NF, Petty NK, ben Zakour NL, Beatson SA. BLAST Ring Image Generator (BRIG): simple prokaryote genome comparisons. BMC Genomics. 2011 Dec 8;12(1):402. doi: 10.1186/1471-2164-12-402 21824423 PMC3163573

[39] Arndt D, Grant JR, Marcu A, Sajed T, Pon A, Liang Y, et al. PHASTER: a better, faster version of the PHAST phage search tool. Nucleic Acids Research. 2016 Jul 8;44(W1):W16–21. doi: 10.1093/nar/gkw387 27141966 PMC4987931

[40] Reis-Cunha JL, Bartholomeu DC, Manson AL, Earl AM, Cerqueira GC. ProphET, prophage estimation tool: A stand-alone prophage sequence prediction tool with self-updating reference database. PLOS ONE. 2019 Oct 2;14(10):e0223364. doi: 10.1371/journal.pone.0223364 31577829 PMC6774505

[41] Darling AE, Mau B, Perna NT. progressiveMauve: Multiple Genome Alignment with Gene Gain, Loss and Rearrangement. PLoS ONE. 2010 Jun 25;5(6):e11147. doi: 10.1371/journal.pone.0011147 20593022 PMC2892488

[42] Alcock BP, Raphenya AR, Lau TTY, Tsang KK, Bouchard M, Edalatmand A, et al. CARD 2020: antibiotic resistome surveillance with the comprehensive antibiotic resistance database. Nucleic Acids Res. 2020;48(D1):D517–25. doi: 10.1093/nar/gkz935 31665441 PMC7145624

[43] Bortolaia V, Kaas RS, Ruppe E, Roberts MC, Schwarz S, Cattoir V, et al. ResFinder 4.0 for predictions of phenotypes from genotypes. Journal of Antimicrobial Chemotherapy. 2020 Dec 1;75(12):3491–500. doi: 10.1093/jac/dkaa345 32780112 PMC7662176

[44] Minh BQ, Schmidt HA, Chernomor O, Schrempf D, Woodhams MD, von Haeseler A, et al. IQ-TREE 2: New Models and Efficient Methods for Phylogenetic Inference in the Genomic Era. Molecular Biology and Evolution. 2020 Aug;37(5):1530–4. doi: 10.1093/molbev/msaa015 32011700 PMC7182206

[45] Kalyaanamoorthy S, Minh BQ, Wong TKF, von Haeseler A, Jermiin LS. ModelFinder: fast model selection for accurate phylogenetic estimates. Nature Methods. 2017 Aug;14(6):587–9. doi: 10.1038/nmeth.4285 28481363 PMC5453245

[46] Hoang DT, Chernomor O, von Haeseler A, Minh BQ, Vinh LS. UFBoot2: Improving the Ultrafast Bootstrap Approximation. Molecular Biology and Evolution. 2018 Aug;35(2):518–22. doi: 10.1093/molbev/msx281 29077904 PMC5850222

[47] Letunic I, Bork P. Interactive tree of life (iTOL) v3: an online tool for the display and annotation of phylogenetic and other trees. Nucleic Acids Research. 2016 Aug;44(W1):W242–5. doi: 10.1093/nar/gkw290 27095192 PMC4987883

[48] Ronquist F, Teslenko M, van der Mark P, Ayres DL, Darling A, Höhna S, et al. MrBayes 3.2: Efficient Bayesian Phylogenetic Inference and Model Choice Across a Large Model Space. Systematic Biology. 2012 Aug;61(3):539–42. doi: 10.1093/sysbio/sys029 22357727 PMC3329765

[49] Marçais G, Delcher AL, Phillippy AM, Coston R, Salzberg SL, Zimin A. MUMmer4: A fast and versatile genome alignment system. PLOS Computational Biology. 2018 Aug;14(1):e1005944. doi: 10.1371/journal.pcbi.1005944 29373581 PMC5802927

[50] de Albuquerque TERRA DA, VILELA EG, SILVA ROS, LEÃO LA, LIMA KS, PASSOS RIFÂ, et al. STRUCTURING A FECAL MICROBIOTA TRANSPLANTATION CENTER IN A UNIVERSITY HOSPITAL IN BRAZIL. Arquivos de Gastroenterologia. 2020 Aug;57(4):434–58. doi: 10.1590/S0004-2803.202000000-79 33331486

[51] Santana JA, Silva BA, Trevizani NAB, Souza AMA e, Lima GMN de, Furtado NRM, et al. Isolation and antimicrobial resistance of coagulase-negative staphylococci recovered from healthy tortoises in Minas Gerais, Brazil. Ciência Rural. 2022;52(7).

[52] Santana JA, Colombo SA, Silva BA, Diniz AN, de Almeida LR, Junior CAO, et al. Clostridioides difficile and multi-drug-resistant staphylococci in free-living rodents and marsupials in parks of Belo Horizonte, Brazil. Brazilian Journal of Microbiology. 2022 Aug;53(1):401–10. doi: 10.1007/s42770-021-00640-x 34761356 PMC8882503

[53] Rosario Medina I, Román Fuentes L, Batista Arteaga M, Real Valcárcel F, Acosta Arbelo F, Padilla del Castillo D, et al. Pigeons and their droppings as reservoirs of Candida and other zoonotic yeasts. Revista Iberoamericana de Micología. 2017 Oct;34(4):211–4. doi: 10.1016/j.riam.2017.03.001 28720316

[54] Silva ROS, Ribeiro MG, de Paula CL, Pires IH, Oliveira Junior CA, Diniz AN, et al. Isolation of Clostridium perfringens and Clostridioides difficile in diarrheic and nondiarrheic cats. Anaerobe. 2020 Apr;62:102164. doi: 10.1016/j.anaerobe.2020.102164 32151948

[55] Griffiths D, Fawley W, Kachrimanidou M, Bowden R, Crook DW, Fung R, et al. Multilocus Sequence Typing of *Clostridium difficile*. Journal of Clinical Microbiology. 2010 Mar;48(3):770–8. 20042623 10.1128/JCM.01796-09 PMC2832416

[56] Imwattana K, Rodríguez C, Riley T v., Knight DR. A species-wide genetic atlas of antimicrobial resistance in Clostridioides difficile. Microbial Genomics. 2021 Nov 18;7(11). doi: 10.1099/mgen.0.000696 34793295 PMC8743556

[57] Freeman J, Bauer MP, Baines SD, Corver J, Fawley WN, Goorhuis B, et al. The Changing Epidemiology of *Clostridium difficile* Infections. Clinical Microbiology Reviews. 2010 Jul;23(3):529–49. 20610822 10.1128/CMR.00082-09 PMC2901659

[58] Knight DR, Elliott B, Chang BJ, Perkins TT, Riley T v. Diversity and Evolution in the Genome of Clostridium difficile. Clinical Microbiology Reviews. 2015 Jul;28(3):721–41. doi: 10.1128/CMR.00127-14 26085550 PMC4475645

[59] Krutova M, Matejkova J, Kuijper EJ, Drevinek P, Nyc O. Clostridium difficile PCR ribotypes 001 and 176 –the common denominator of C. difficile infection epidemiology in the Czech Republic, 2014. Eurosurveillance. 2016 Aug;21(29). doi: 10.2807/1560-7917.ES.2016.21.29.30296 27484171

[60] Razavi B, Apisarnthanarak A, Mundy LM. Clostridium difficile: Emergence of Hypervirulence and Fluoroquinolone Resistance. Infection. 2007 Oct 20;35(5):300–7. doi: 10.1007/s15010-007-6113-0 17885732

[61] Gupta A, Cifu AS, Khanna S. Diagnosis and Treatment of *Clostridium difficile* Infection. JAMA. 2018 Sep 11;320(10):1031. 30178042 10.1001/jama.2018.12194

[62] Bandelj P, Golob M, Ocepek M, Zdovc I, Vengust M. Antimicrobial Susceptibility Patterns of *Clostridium difficile* Isolates from Family Dairy Farms. Zoonoses and Public Health. 2017 May;64(3):213–21. 27484050 10.1111/zph.12299

[63] Pirš T, Avberšek J, Zdovc I, Krt B, Andlovic A, Lejko-Zupanc T, et al. Antimicrobial susceptibility of animal and human isolates of Clostridium difficile by broth microdilution. Journal of Medical Microbiology. 2013 Sep 1;62(9):1478–85. doi: 10.1099/jmm.0.058875-0 23861298

[64] Carter GP, Douce GR, Govind R, Howarth PM, Mackin KE, Spencer J, et al. The Anti-Sigma Factor TcdC Modulates Hypervirulence in an Epidemic BI/NAP1/027 Clinical Isolate of Clostridium difficile. PLoS Pathogens. 2011 Oct 13;7(10):e1002317. doi: 10.1371/journal.ppat.1002317 22022270 PMC3192846

[65] Matamouros S, England P, Dupuy B. Clostridium difficile toxin expression is inhibited by the novel regulator TcdC. Molecular Microbiology. 2007 May 30;64(5):1274–88. doi: 10.1111/j.1365-2958.2007.05739.x 17542920

[66] Stabler RA, He M, Dawson L, Martin M, Valiente E, Corton C, et al. Comparative genome and phenotypic analysis of Clostridium difficile 027 strains provides insight into the evolution of a hypervirulent bacterium. Genome Biology. 2009;10(9):R102. doi: 10.1186/gb-2009-10-9-r102 19781061 PMC2768977

[67] Dupuy B, Govind R, Antunes A, Matamouros S. Clostridium difficile toxin synthesis is negatively regulated by TcdC. Journal of Medical Microbiology. 2008 Jun 1;57(6):685–9. doi: 10.1099/jmm.0.47775-0 18480323

[68] Spigaglia P, Mastrantonio P. Molecular Analysis of the Pathogenicity Locus and Polymorphism in the Putative Negative Regulator of Toxin Production (TcdC) among *Clostridium difficile* Clinical Isolates. Journal of Clinical Microbiology. 2002 Sep;40(9):3470–5. 12202595 10.1128/JCM.40.9.3470-3475.2002 PMC130716

[69] Stabler RA, Dawson LF, Valiente E, Cairns MD, Martin MJ, Donahue EH, et al. Macro and Micro Diversity of Clostridium difficile Isolates from Diverse Sources and Geographical Locations. PLoS ONE. 2012 Aug;7(3):e31559. doi: 10.1371/journal.pone.0031559 22396735 PMC3292544

[70] Shen E, Zhu K, Li D, Pan Z, Luo Y, Bian Q, et al. Subtyping analysis reveals new variants and accelerated evolution of Clostridioides difficile toxin B. Communications Biology. 2020 Dec 3;3(1):347. doi: 10.1038/s42003-020-1078-y 32620855 PMC7335066

[71] Carter GP, Rood JI, Lyras D. The role of toxin A and toxin B in the virulence of Clostridium difficile. Trends in Microbiology. 2012 Jan;20(1):21–9. doi: 10.1016/j.tim.2011.11.003 22154163

[72] Rodriguez C, van Broeck J, Taminiau B, Delmée M, Daube G. Clostridium difficile infection: Early history, diagnosis and molecular strain typing methods. Microbial Pathogenesis. 2016 Aug;97:59–78. doi: 10.1016/j.micpath.2016.05.018 27238460

[73] Douce G, Goulding D. Refinement of the Hamster Model of Clostridium difficile Disease. In 2010. p. 215–27. 10.1007/978-1-60327-365-7_14 20597012

[74] Keel MK, Songer JG. The Comparative Pathology of *Clostridium difficile* -associated Disease. Veterinary Pathology. 2006 May 26;43(3):225–40. 16672570 10.1354/vp.43-3-225

[75] Buckley AM, Spencer J, Candlish D, Irvine JJ, Douce GR. Infection of hamsters with the UK Clostridium difficile ribotype 027 outbreak strain R20291. Journal of Medical Microbiology. 2011 Aug 1;60(8):1174–80. doi: 10.1099/jmm.0.028514-0 21330415 PMC3167879

[76] Vitucci JC, Pulse M, Tabor-Simecka L, Simecka J. Epidemic ribotypes of Clostridium (now Clostridioides) difficile are likely to be more virulent than non-epidemic ribotypes in animal models. BMC Microbiology. 2020 Aug;20(1):27. doi: 10.1186/s12866-020-1710-5 32024477 PMC7003423

[77] Vohra P, Poxton IR. Comparison of toxin and spore production in clinically relevant strains of Clostridium difficile. Microbiology (N Y). 2011 May 1;157(5):1343–53. doi: 10.1099/mic.0.046243-0 21330434

[78] Castro-Córdova P, Mora-Uribe P, Reyes-Ramírez R, Cofré-Araneda G, Orozco-Aguilar J, Brito-Silva C, et al. Entry of spores into intestinal epithelial cells contributes to recurrence of Clostridioides difficile infection. Nature Communications. 2021 Dec 18;12(1):1140. doi: 10.1038/s41467-021-21355-5 33602902 PMC7893008

[79] Roxas BAP, Roxas JL, Claus-Walker R, Harishankar A, Mansoor A, Anwar F, et al. Phylogenomic analysis of Clostridioides difficile ribotype 106 strains reveals novel genetic islands and emergent phenotypes. Scientific Reports. 2020 Aug;10(1):22135. doi: 10.1038/s41598-020-79123-2 33335199 PMC7747571

[80] Suárez-Bode L, Barrón R, Pérez JL, Mena A. Increasing prevalence of the epidemic ribotype 106 in healthcare facility–associated and community-associated Clostridioides difficile infection. Anaerobe. 2019 Aug;55:124–9. doi: 10.1016/j.anaerobe.2018.12.002 30550807

[81] Carlson TJ, Blasingame D, Gonzales-Luna AJ, Alnezary F, Garey KW. Clostridioides difficile ribotype 106: A systematic review of the antimicrobial susceptibility, genetics, and clinical outcomes of this common worldwide strain. Anaerobe. 2020 Apr;62:102142. doi: 10.1016/j.anaerobe.2019.102142 32007682 PMC7153973

[82] de Oliveira CA, de Paula Gabardo M, Guedes RMC, Poncet F, Blanc DS, Lobato FCF, et al. Rodents are carriers of Clostridioides difficile strains similar to those isolated from piglets. Anaerobe. 2018 Jun;51:61–3. doi: 10.1016/j.anaerobe.2018.04.006 29680295

[83] Diniz AN, Cruz DSG, Ramos CP, Oliveira Júnior CA, Winter IC, Lima JTB de, et al. Clostridioides (Clostridium) difficile-associated diarrhea in equine in Minas Gerais, Brazil: clinical and microbiological characterization of six cases. Ciência Rural. 2021;51(8).

[84] Lim SC, Knight DR, Riley TV. Clostridium difficile and One Health. Clinical Microbiology and Infection. 2020 Jul;26(7):857–63. doi: 10.1016/j.cmi.2019.10.023 31682985

[85] Janezic S, Zidaric V, Pardon B, Indra A, Kokotovic B, Blanco JL, et al. International Clostridium difficile animal strain collection and large diversity of animal associated strains. BMC Microbiology. 2014 Dec 28;14(1):173. doi: 10.1186/1471-2180-14-173 24972659 PMC4100527

[86] Goorhuis A, Bakker D, Corver J, Debast SB, Harmanus C, Notermans DW, et al. Emergence of *Clostridium difficile* Infection Due to a New Hypervirulent Strain, Polymerase Chain Reaction Ribotype 078. Clinical Infectious Diseases. 2008 Nov;47(9):1162–70. 18808358 10.1086/592257

[87] Knetsch CW, Connor TR, Mutreja A, van Dorp SM, Sanders IM, Browne HP, et al. Whole genome sequencing reveals potential spread of Clostridium difficile between humans and farm animals in the Netherlands, 2002 to 2011. Eurosurveillance. 2014 Nov 13;19(45). doi: 10.2807/1560-7917.es2014.19.45.20954 25411691 PMC4518193

[88] Martin JSH, Monaghan TM, Wilcox MH. Clostridium difficile infection: epidemiology, diagnosis and understanding transmission. Nature Reviews Gastroenterology & Hepatology. 2016 Apr 9;13(4):206–16. doi: 10.1038/nrgastro.2016.25 26956066

