# Supplementary material for: Characterization of the virulence of three novel clade 2 Clostridioides (Clostridium) difficile strains and a two-year screening in animals and humans in Brazil
Source: PLoS One. 2022 Aug 26;17(8):e0273013. doi: 10.1371/journal.pone.0273013 (PMC9416996; doi:10.1371/journal.pone.0273013)
Supplement: S3 File — (PDF) [file pone.0273013.s003.pdf]

Supplementary Table 1: Virulence genes and their function in the Brazilian *Clostridioides difficile* strains RT883, RT884 and RT885.

Genes	Function/ Relevance	Strains		
		RT883	RT884	RT885
fbpc8	Adhesin	+	+	+
cwp66	Adhesin	+	+	+
cwp84	Adhesin	+	+	+
slpa	Adhesin	+	+	+
groel	Adhesin	+	+	+
cfr	Linezolid Resistance	+	+	+
rpld	Linezolid Resistance	+	-	+
vang	Vancomycin resistance	+	+	+
vans	Vancomycin resistance	+	-	-
ermb	Erythromycin resistance	-	-	-
soda	Superoxide dismutase	-	-	-
trea	Trehalose metabolism	-	-	-
flic	Flagellin	-	-	-
flid	Flagellin	+	+	+
spo0a	Sporulation	+	+	+
cotaf	Spore formation	+	+	+
cotbf	Spore protein	+	+	+
cotcf	Spore protein	+	+	+

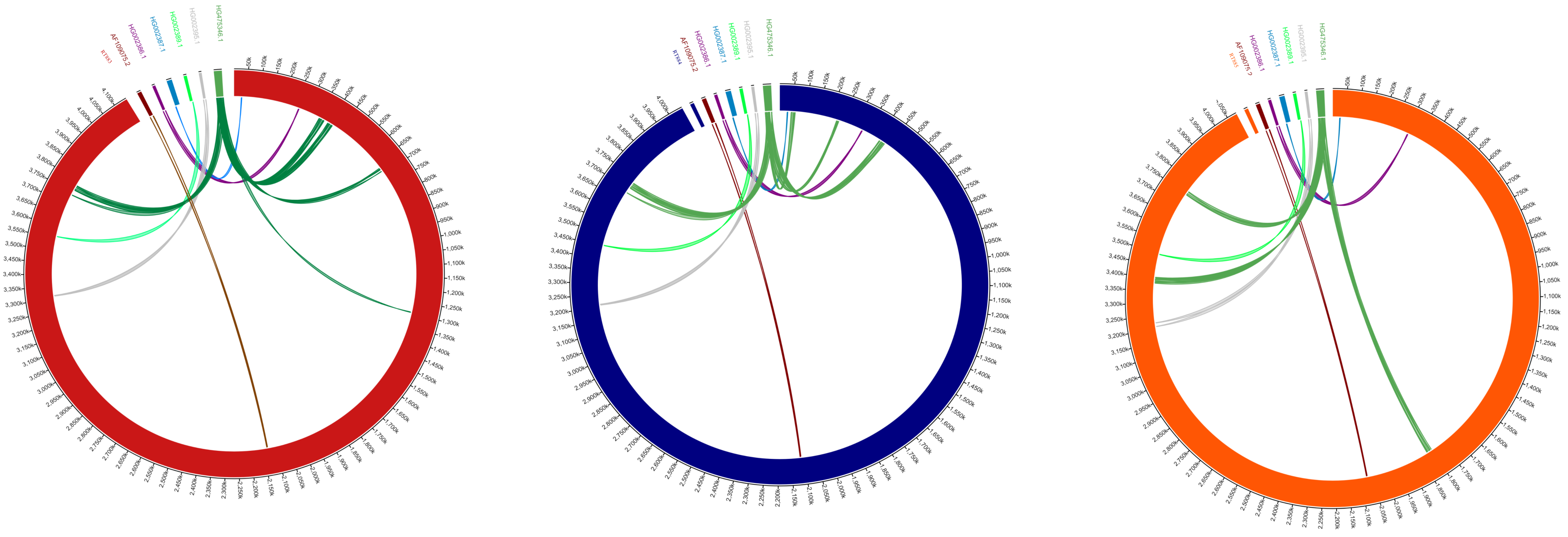

Supplementary Figure 1: Analysis of transposons regions detected on the Brazilian isolates RT883, 884 and 885. RT883 (red); RT884 (blue); RT885 (orange). Part of the following *C. difficile* transposons were detected in the three Brazilian strains: AF109075, HG475346, KC166248, HG002387, HG002396, HG002395, HG002389, HG002386, AF333235, AF226276.

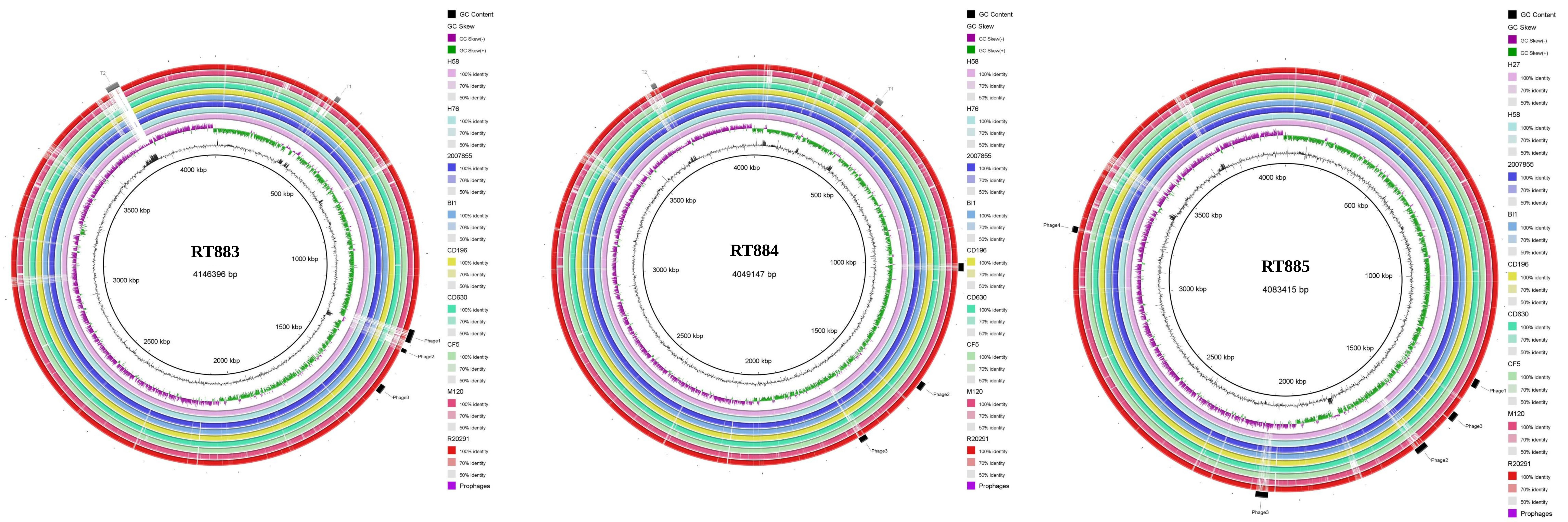

Supplementary Figure 2: Circular representations of *Clostridioides difficile* genomes generated with BRIG software. Graphical circular map of *C. difficile* from RT883, RT884, RT885 genome. From outside to the center: predicted phage regions by PHAST; reference strains R20291, M68, CF5, 2007855, M120, CD196, BI1 and CD630; GC skew; and, GC content.

Supplementary table 2: Alleles from relevant genes (*tcdB*, *tcdC*, *gyrA* and *gyrB*) of three novel Brazilian clade 2 strains (RT883, RT884 and RT885).

Gene	Function/Relevance	Strains			Comments
		RT883	RT884	RT885	
tcdB	Toxin B	+ Allele 23*	+ Allele 8	+ Allele10	
tcdC	Negative regulator of toxin gene expression	+ Allele 16	+ Allele 57*	+ Allele 8	Expression affected
gyrA	Fluorquinolone resistance	Allele 76 Thr-82	Allele 76 Thr-82	Allele 76 Thr-82	No typical fluoroquinolone resistance mutation
gyrB		Allele 57 Thr-82	Allele 57 Thr-82	Allele 57 Thr-82	

*Novel alleles identified in the present study

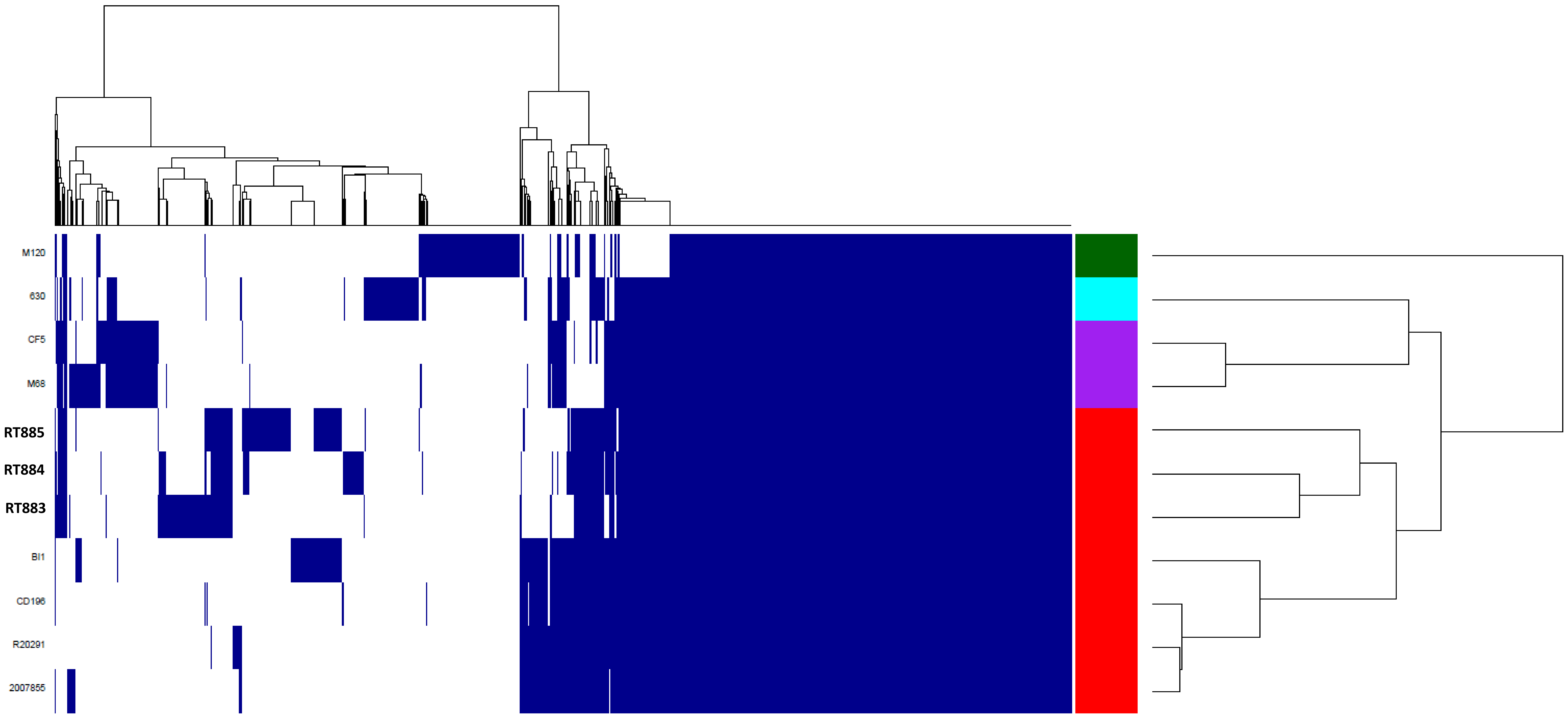

Supplementary Figure 3: Pan-genome and core-genome content of *C. difficile* isolates. (A) In this image, each line corresponds to a *C. difficile* isolate/strain, and each column to a gene. Gene presence/absence is represented respectively by blue and white boxes. The dendrograms in the X and Y axis correspond to the hierarchical clustering of respectively the isolate and gene patterns. The color strip represents the *C. difficile* Clades, where Clade 1, 2, 3 and 5 are represented by respectively cyan, red, purple and green.

Supplementary table 3: Antimicrobial susceptibility of the three novel Brazilian clade 2 strains (RT883, RT884 and RT885) by ETEST (bioMérieux Brazil, France)

Antimicrobial	RT883	RT884	RT885
		(mg/mL)	
**Metronidazole**	0,25	0,25	0,25
**Vancomycin**	0,25	0,25	1,0
**Erythromycin**	0,75	0,75	0,75
**Clindamycin**	2,0	4,0	8,0
**Moxifloxacin**	2,0	2,0	6,0
**Tetracycline**	0,047	0,094	0,094
**Rifampin**	0,002	0,002	0,002

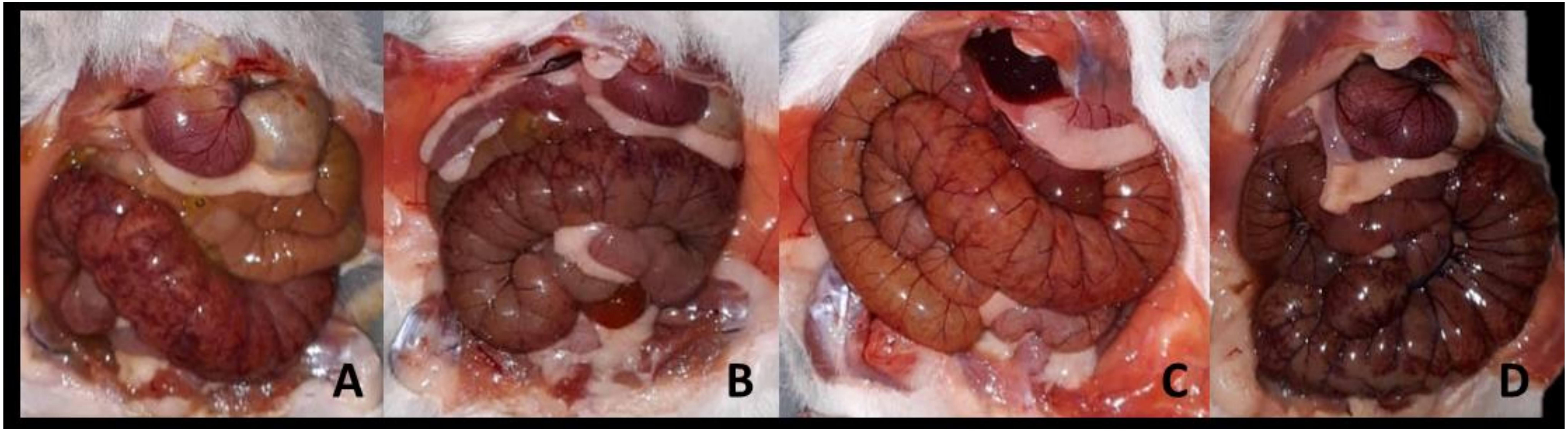

Supplementary Figure 4: Abdominal viscera, adult Syrian hamster (*Mesocricetus auratus*) *C. difficile* infection (CDI) caused by a reference RT027 strain (A), the Brazilian isolates RT883 (B), 884 (C) and 885 (D). In all, the cecum is hyperemic and shows multiple ecchymotic hemorrhages, typical of CDI in this species (Keel and Songer, 2006).
